# Chaperonin TRiC/CCT subunit CCT7 is involved in the replication of canine parvovirus in F81 cells

**DOI:** 10.3389/fmicb.2024.1346894

**Published:** 2024-02-07

**Authors:** Xia Su, Hongzhuan Zhou, Fuzhou Xu, Jin Zhang, Bing Xiao, Qi Qi, Lulu Lin, Bing Yang

**Affiliations:** Beijing Key Laboratory for Prevention and Control of Infectious Diseases in Livestock and Poultry, Institute of Animal Husbandry and Veterinary Medicine, Beijing Academy of Agriculture and Forestry Sciences, Beijing, China

**Keywords:** canine parvovirus, CCT7, VP2, replication, stability

## Abstract

Canine parvovirus (CPV) is one of the most common lethal viruses in canines. The virus disease is prevalent throughout the year, with high morbidity and mortality rate, causing serious harm to dogs and the dog industry. Previously, yeast two hybrid method was used to screen the protein chaperonin containing TCP-1 (CCT7) that interacts with VP2. However, the mechanism of interactions between CCT7 and VP2 on CPV replication remains unclear. In this study, we first verified the interaction between CCT7 and viral VP2 proteins using yeast one-to-one experiment and co-immunoprecipitation (CoIP) experiment. Laser confocal microscopy observation showed that CCT7 and VP2 were able to co-localize and were mostly localized in the cytoplasm. In addition, the study of VP2 truncated mutant found that the interaction region of VP2 with CCT7 was located between amino acids 231 and 320. Cycloheximide (CHX) chase experiments showed that CCT7 can improve the stability of VP2 protein. After further regulation of CCT7 expression in F81 cells, it was found that the expression level of VP2 protein was significantly reduced after knocking down CCT7 expression by RNA interference (RNAi) or HSF1A inhibitor, and increased after overexpressing host CCT7. The study reveals the role of VP2 interacting protein CCT7 in the replication process of CPV, which could provide a potential target for the prevention and control of CPV.

## 1 Introduction

Canine parvovirus (CPV) is one of the main enteric pathogens causing gastroenteritis in dogs, and its typical clinical manifestations include vomiting, hemorrhagic diarrhea and dehydration. It is an acute and fatal infectious disease in puppies (Nandi and Kumar, 2010; Tuteja et al., 2022), with a wide range of hosts (Canuti et al., 2020). The CPV genome substitution rate is similar to that of RNA viruses (Carmichael and Binn, 1981), leading to the continuous emergence of new mutant strains. With gradual mutation, the original CPV-2 type has been replaced by five subtypes: CPV-2a, CPV-2b, New CPV-2a, New CPV-2b, and CPV-2c (Hao et al., 2022). All subtypes have been reported in China (Qi et al., 2020). CPV is a single-stranded, negative-stranded, naked DNA virus without envelope, which contains two open reading frames (ORFs), encoding non-structural proteins (NS1 and NS2) and structural proteins (VP1 and VP2), respectively (Miranda and Thompson, 2016; de Oliveira Santana et al., 2022). VP2 protein is one of the important components in the viral capsid of CPV. Moreover, it contains the neutralizing antigen domains of CPV, which can induce the neutralizing antibodies *in vivo*. In addition, VP2 also contains key sites that determine the host range and binding sites for transferrin receptors on the host cell membrane, which can mediate the infection of viral particles (Allison et al., 2016).

After the virus infects cells, it is necessary to utilize the translation mechanism of the host cell to synthesize a large number of proteins required for viral proliferation, and use the host companion network to fold viral peptides (Knowlton et al., 2021). Among them, the eukaryotic chaperonin T-complex protein-1 (TCP-1) ring complex (TRiC), also known as CCT (chaperonin containing TCP-1), plays an important role in folding proteins related to apoptosis and cell cycle (Knowlton et al., 2018; Wang et al., 2020). The TRiC/CCT chaperonin complex is approximately 1000 kDa in size, and the complex is composed of eight distinct subunits called CCTα, β, γ, δ, ε, ζ, η, and θ, which correspond to CCT1-8 in yeast (Wang et al., 2020). TRiC/CCT chaperonin is located in the nucleus and cytosol (Tahmaz et al., 2021). In addition, TRiC/CCT chaperonin can also directly or indirectly participate in the infection and regulation of a variety of viruses, such as hepatitis C virus (HCV), Fowl adenovirus serotype 4 (FAdV-4) and influenza A virus (IAV) (Inoue et al., 2011; Gao et al., 2019; Zhang et al., 2020). However, the role of TRiC/CCT chaperone protein in the proliferation and infection of CPV and its related molecular mechanisms have not been reported yet.

In this study, we verified the interaction and co-localization between CCT7 and viral VP2 proteins. In addition, we identified the region in VP2 that interact with CCT7 by constructing VP2 truncation mutants. Cycloheximide (CHX) chase experiment was used to detect the effect of CCT7 on VP2 stability. Small RNA interference (RNAi), HSF1A inhibitor and overexpressing plasmid were further used to evaluate the impact of changes in CCT7 expression levels on CPV replication. This study reveals the role of VP2 interacting protein CCT7 in the replication process of CPV.

## 2 Materials and methods

### 2.1 Cell and virus

F81 cell line was previously purchased from the American Type Culture Collection (ATCC) and has been kept in our lab ever since. The growth medium was composed of DMEM (Gibco, NY, USA), 10% fetal bovine serum (Gibco, NY, USA), 100 U/mL penicillin and 0.1 mg/mL streptomycin. The cell culture temperature was 37*^Circ^*C and the CO_2_ concentration was 5%. The New CPV-2a strain SD6 (297Ala, 426Asn) was also isolated and preserved in our laboratory, and its VP2 coding gene was registered in GenBank as MN101724.

### 2.2 Reagents and antibodies

Anti-Parvovirus primary Antibody (CPV1-2A1) was purchased from Santa Cruz (Santa Cruz Biotechnology, TX, USA). p3xFlag-CMV-14, pCMV-Myc, pDsRed-monomer-N1 and pEGFP-N1 was purchased from YouBio (YouBio Technology, Hunan, China). T4 DNA ligase (M0202), *Xho*I (R0146), *Bam*HI (R0136), *Eco*RI (R3101), *Kpn*I (R3142), *Sal*I (R0138) were purchased from New England Biolabs (NEB, MA, USA). EndoFree Midi Plasmid Kit (DP108) and SuperReal PreMix Plus (FP205) were purchased from TIANGEN (TIANGEN BIOTECH, Beijing, China). Y2HGold Yeast Strain was purchased from Takara (Takara Biomedical Technology, Beijing, China). CCT7 Rabbit polyclonal antibody (A12146) and GAPDH Rabbit polyclonal antibody (AC001) were purchased from ABclonal (ABclonal Technology, Hubei, China). Beta-actin monoclonal antibody (AC-15), Lipofectamine™3000, Lipofectamine™2000, Lipofectamine™RNAiMAX, DAPI (4′,6-Diamidino-2-Phenylindole, Dihydrochloride D1306), Goat anti-Rabbit IgG (H+L) Secondary Antibody, DyLight™ 488 (35552), Goat anti-Mouse IgG (H+L) Cross-Adsorbed Secondary Antibody, TRITC(A16077), SuperSignal™ West Pico PLUS (34580), protein A/G (80104) were purchased from Thermo Fisher (Thermo Fisher Scientific, MA, USA). ANTI-FLAG^®^ M2 (F1804) antibody, Anti-c-Myc (M4439) antibody, Triton X-100(X-100) and cycloheximide (CHX, C7698) solution were purchased from Sigma (Sigma-Aldrich, Saint Louis, USA). Anti-c-Myc Magnetic Beads (HY-K0206), Anti-Flag Affinity Gel (HY-K0217), and Anti-Flag Magnetic Beads (HY-K0207) were purchased from MedChemExpress (MedChemExpress, NJ, USA).

### 2.3 Verification through yeast one-to-one hybridization

pGBKT7-VP2 was co-transformed with the prey plasmids pGADT7-CCT7 into Y2HGold competent yeast cells, and the positive control (pGBKT7-p53+pGADT7-T) and the negative control (pGBKT7-lam+pGADT7-T) were prepared at the same time. After transformation, obtained transformation mixture was plated onto SD/-Trp-Leu, SD/-Trp-Leu/X-α-gal and SD/-Trp-Leu-His-Ade/X-α-gal/AbA agar plates, respectively. Then these plates were incubated for 3–5 days at 30°C to observe colony growth and color change.

### 2.4 Construction of expression plasmids

Based on the gene sequence of cat CCT7 (Gene ID: 101080777), primers were designed to amplify the CCT7 gene from F81 cells, and different enzyme restriction sites were added at both ends of the primers, the specific sequences were listed in Table 1. RNA was extracted from F81 cells using the Qiagen RNeasy Mini Kit (Qiagen, Hilden, Germany) and reverse transcribed into cDNA for further use. After amplification and cloning, the correct sequence fragments containing different restriction endonuclease sites were identified by sequencing, and cloned into different expression vectors to construct pDsRed-monomer-N1-CCT7, p3xFlag-CMV-14-CCT7 and pCMV-Myc-CCT7 expression plasmids.

**TABLE 1:** Primers used in this study.

Primers^a^	Sequences (5′-3′)^b^	Restriction site
Myc-CCT7-F	CCGAATTCACATGATGCCCACACCAGTTAT	*Eco*RI
Myc-CCT7-R	ACCTCGAGGGTGGGGGCGGCCCCGGCCCCG	*Xho*I
Flag-CCT7-F	CTGAATTCCATGATGCCCACACCAGTTATC	*Eco*RI
Flag-CCT7-R	GAGGTACCGAGTGGGGGCGGCCCCGGCCCCG	*Kpn*I
DsRed-CCT7-F	GACTCGAGATGATGCCCACACCAGTTAT	*Xho*I
DsRed-CCT7-R	GCGAATTCGGTGGGGGCGGCCCCGGCCCCG	*Eco*RI
EGFP-VP2-F	GACTCGAGCTATGAGTGATGGAGCAGTTCAACC	*Xho*I
EGFP-VP2-R	GCGGATCCTTAGTATAATTTTCTAGGTGC	*Bam*HI
Myc-VP2-F	CGGTCGACAATGGAGCAGAAGCTGATCTCAGAG GAGGACCTGATGAGTGATGGAGCAGTTCA	*Sal*I
Myc-VP2-R	CCGGTACCTTAGTATAATTTTCTAGGTGCTAGT	*Kpn*I
Flag-VP2-F	GCGAATTCCATGAGTGATGGAGCAGTTCAA	*Eco*RI
Flag-VP2-R	CTGGTACCGTGTATAATTTTCTAGGTGCTA	*Kpn*I
Myc-VP2-1-500-R	GCGGTACCTTATACAAATAATTGACCAGGACAA	*Kpn*I
Myc-VP2-1-410-R	GCGGTACCTTATGGATATCTTCCTGTATCTTGA	*Kpn*I
Myc-VP2-1-320-R	GCGGTACCTTATCCCATTTGAGTTACACCACGT	*Kpn*I
Myc-VP2-1-230-R	GCGGTACCTTATGTTGGTGTGCCACTAGTTCCA	*Kpn*I
Myc-VP2-321-585-F	CGGTCGACAATGGAGCAGAAGCTGATCTCAGAGGAGGACCTGAATACAAACATTATTACTGA	*Sal*I
Myc-VP2-231-585-F	CGGTCGACGATGGAGCAGAAGCTGATCTCAGAGGAGGACCTGAATATATACCATGGTACAGA	*Sal*I
Myc-VP2-141-585-F	CGGTCGACAATGGAGCAGAAGCTGATCTCAGAGGAGGACCTGTTTGAACAAGAAATTTTTAA	*Sal*I
Myc-VP2-585-R	GCGGTACCTTAGTATAATTTTCTAGGTGCT	*Kpn*I
VP2-F	CAAATAGAGCATTGGGCTTACC	
VP2-R	TCCCATTTGAGTTACACCACG	
CCT7-F	GCTGGCGTCGCGTTCAAGAAG	
CCT7-R	TTGCCTGATAATCCTCAACTGTG	
β-actin-F	CATGTACGTGGCCATCCAGGC	
β-actin-R	CTCCTTGATGTCACGCACAAT	

*^a^*F denotes forward PCR primer; R denotes reverse PCR primer.

*^b^*Restriction sites are underlined.

According to the molecular analysis of the full-length VP2 gene (Silva et al., 2022) and its gene sequence (MN101724), primers for the full-length and truncated fragments of the VP2 gene were designed, with the corresponding restriction enzyme sites added at both ends of the primers (Table 1). Total DNA was extracted from CPV-infected F81 cells for PCR template, after amplification and cloning, the correct fragments identified by sequencing were inserted into various expression vectors to construct the following recombinant plasmids p3xFlag-CMV-14-VP2, pCMV-Myc-VP2, pEGFP-N1-VP2, pCMV-Myc-VP2-1-500, pCMV-Myc-VP2-1-410, pCMV-Myc-VP2-1-320, pCMV-Myc-VP2-1-230, pCMV-Myc-VP2-321-585, pCMV-Myc-VP2-231-585 and pCMV-Myc-VP2-141-585.

### 2.5 Quantitative PCR

Cells transfected with small interfering RNAs or overexpression plasmids or added with HSF1A inhibitor were frozen and thawed three times after incubation for the required time. Then samples were stored at −80°C for future use. When absolute quantitative PCR (qPCR) was used to determine the virus copy number, DNA was extracted with Quick DNA Extraction Kit (Cwbio, Jiangsu, China). The pMD-VP2S positive plasmid stored in the laboratory was used as a template, and a quantitative standard curve was prepared according to the previously reported method (Zhou et al., 2019). The sample was quantified using the previously reported primers for amplifying VP2 (Table 1), and the DNA copy number of the VP2-encoding gene in the sample was determined based on the resulting standard curve. The reagents used for quantification are all SuperReal PreMix Plus (SYBR Green) (TIANGEN, Beijing, China), and the reaction system is configured according to the instructions provided by the manufacturer. Amplification was completed on the CFX Connect™ Real-Time PCR Detection System (Bio-Rad, CA, USA) instrument.

### 2.6 Western blot

Total protein was extracted using the ProteinExt^®^ Mammalian Total Protein Extraction Kit (TransGen Biotech, Beijing, China). Briefly, the collected cells were lysed for 20 min on ice, and then centrifuged at 14,000 rpm at 4°C for 20 min. The protein concentration of collected supernatant was quantified with Pierce™ BCA Protein Assay Kit (Thermo Scientific, MA, USA). After running SDS-PAGE electrophoresis, the proteins in the gel were transferred to PVDF membranes (Millipore, MA, USA) and blocked with 5% skim milk. The blocked PVDF membrane was incubated with the primary antibodies and secondary antibodies against the target fragments, detected using SuperSignal™ West Pico PLUS Chemiluminescent Substrate Kit (Thermo Scientific, MA, USA), and images were obtained using Amersham Imager 600 (GE Healthcare, IL, USA). Band intensities were measured by Image J software.

### 2.7 Immunoprecipitation

To determine of the interaction and interaction region between VP2 and CCT7. F81 cells were inoculated onto 6-well cell culture plates, and after the cells grew to about 60% confluence, the wild-type plasmids pCMV-Myc-VP2 or truncated mutants pCMV-Myc-VP2-1-500, pCMV-Myc-VP2-1-410, pCMV-Myc-VP2-1-320, pCMV-Myc-VP2-1-230, pCMV-Myc-VP2-321-585, pCMV-Myc-VP2-231-585 and pCMV-Myc-VP2-141-585 were co-transfected into F81 cells with p3xFlag-CMV-14-CCT7, respectively, using Lipofectamine™3000 (Thermo Fisher Scientific, MA, USA) transfection reagent. The two plasmids were mixed at a concentration of 1:1, and the total amount of plasmids per well was 2 μg. 24 h after transfection, the culture medium was discarded, and then cells were lysed with radio-immunoprecipitation assay (RIPA) lysis buffer with cocktail (MedChemExpress, NJ, USA), and immunoprecipitation was performed with Anti-Flag Magnetic Beads and Anti-c-Myc Magnetic Beads (MedChemExpress, NJ, USA), respectively, according to the instructions. Subsequently, western blot was performed using Anti-Flag M2 antibody (Sigma-Aldrich, Saint Louis, USA) and Anti-c-Myc antibody (Sigma-Aldrich, Saint Louis, USA).

For endogenous interaction assay, F81 cells were inoculated onto 6-well cell culture plates and infected with CPV (MOI = 0.1). The culture medium was discarded after 24 h of infection, and then the cells were lysed by RIPA lysis buffer mentioned above. The obtained lysate was mixed with Pierce Protein A/G Plus Agarose (Thermo Fisher Scientific) combined with Anti-CCT7 (ABclonal Technology, Hubei, China) or IgG (ABclonal Technology, Hubei, China) at 4°C and incubated overnight. After washing, the samples were boiled with 1 × SDS loading buffer for 10 min, and then detected by immunoblotting with Anti-Parvovirus primary Antibody (CPV1-2A1) (Santa Cruz Biotechnology, TX, USA) and anti-CCT7 Rabbit polyclonal antibody (ABclonal Technology, Hubei, China).

### 2.8 Confocal laser scanning microscopy detection

F81 cells were seeded in 24-well cell culture plates with pre-placed glass cover slide, and the cell samples were divided into 3 groups when the cells had grown to about 60% confluence. The first group was co-transfected with pEGFP-VP2 and pDsRed-CCT7 (2 μg, the ratio of two plasmids is 1:1), the second group was infected with CPV 12 h after transfection with pDsRed-CCT7 (2 μg) plasmid, and the third group of F81 cells were directly infected with CPV (MOI = 0.1). The supernatant was discarded at 24 h after transfection and/or infection. Cell samples were fixed with 4% paraformaldehyde (Solarbio, Beijing, China) for 30 min, then permeabilized with 2% bovine serum albumin (BSA) solution (Sigma-Aldrich, Saint Louis, USA) containing 0.1% Triton X-100 for 10 min at room temperature, and further blocked with 2% BSA for 1 h. After blocking, the transfected cells were washed with PBS, and then DAPI (Thermo Fisher Scientific, MA, USA) was added for nuclear staining. For cells infected with CPV, after washing with PBS, cells were incubated at room temperature for 1 h with a mixture of mouse anti VP2 and rabbit anti CCT7 antibodies, then incubated at room temperature with mixed DyLight™ 488 and TRITC coupled secondary antibody solutions for 1 h, and finally stained with DAPI (Thermo Fisher Scientific, MA, USA). Images were collected on a confocal microscope (LSM900, Carl Zeiss, Germany).

### 2.9 RNA interference

Based on the cat CCT7 gene sequence (Gene ID: 101080777), the interference sequences were designed and synthesized by GenePharma (GenePharma, Shanghai, China) to knock down CCT7 expression in F81 cells. The interference and control sequences were RNAi-272 (sense 5′-GGACAAGCUGA UCGUAGAUTT-3′, antisense 5′-AUCUACGAUCAGCUUGUCC TT-3′); RNAi-347 (sense 5′-GGAUUACUCAAGGACCGUU TT-3′, antisense 5′-AACGGUCCUUGAGUAAUCCTT-3′); RNAi- 1400 (5′-GGACAUUGCCAAGUCCCAATT-3′, antisense 5′-UUGGGACUUGGCAAUGUCCTT-3′); negative control (sense 5′-UUCUCCGAACGUGUCACGUTT-3′, antisense 5′-ACGUG ACACGUUCGGAGAATT-3′), respectively. When F81 cells grew to approximately 60% confluence in the 6-well cell culture plates, siRNA and negative control siRNA (siNC) with a concentration of 30 μM was transfected into cells using Lipofectamine™RNAiMAX (Thermo Fisher Scientific, MA, USA), respectively, according to the manufacturer’s instructions (first transfection). In order to better knockdown CCT7, a second transfection using the same method was performed 12 h after the first transfection, and the cells were collected 24 h after the second transfection, followed by subsequent experiments.

### 2.10 CHX chase assay

Protein synthesis inhibition assay was performed with cycloheximide (CHX) (Sigma-Aldrich, Saint Louis, USA). F81 cells were inoculated on a 6-well cell culture plate, and when the cells grew to about 60% confluence, Lipofectamine™3000 (Thermo Fisher Scientific, MA, USA) transfection reagent was used to synchronously transfect p3xFlag-CMV-14-VP2 and pCMV-Myc-CCT7, as well as the control empty vector. The two plasmids were mixed at a concentration of 1:1, and the total amount of plasmids per well was 2 μg. After transfection, cells were incubated for another 20 h, followed by the addition of the same concentration of CHX (CHX, 50 μg/mL). During this period, cells transfected with pCMV-Myc-CCT7 plasmid and corresponding cells transfected with empty vector control were collected at 0, 1, 2, 3, 4 and 5 h after CHX treatment. Finally, the collected samples were subjected to immunoblotting.

F81 cells were inoculated onto a 6-well cell culture plates, and when the cells grew to about 60% confluence, Lipofectamine™RNAiMAX (Thermo Fisher Scientific, MA, USA) transfection reagent was used to transfect si-CCT7 into F81 cells (first transfection). After 12 h of first transfection, Lipofectamine™2000 (Thermo Fisher Scientific, MA, USA) was then used to simultaneously transfect interfering si-CCT7 and p3xFlag-CMV-14-VP2 plasmid into F81 cells (second transfection). After the second round transfection, cells were incubated for another 20 h, followed by the addition of the same concentration of CHX (CHX, 50 μg/mL). During this period, cells transfected with si-CCT7 and corresponding cells transfected with si-NC control were collected at 0, 1, 1.5, 2.0, 2.5 and 3.0 h after CHX treatment. Finally, the collected samples were subjected to immunoblotting assay.

### 2.11 The impact of CCT7 on CPV replication

Small interfering RNA (siRNA), HSF1A inhibitor and overexpression plasmid were used to regulate the expression of CCT7 to investigate its effect on CPV replication. In RNA interference experiment, when F81 cells grew to about 60% confluences, after two rounds interference as mentioned above, CPV (MOI = 0.1) was inoculated 12 h after the second round interference. In the HSF1A inhibitor treatment experiment, F81 cells were treated with HSF1A inhibitors (25, 50 μM) while being inoculated with CPV (MOI = 0.1), after 8 h, fresh culture medium was replaced to continue cultivation. In the overexpression of CCT7 experiment, F81 cells were transfected with pCMV-Myc-CCT7 (2 μg) plasmid using lipo3000 (Thermo Fisher Scientific, MA, USA) and then infected with CPV (MOI = 0.1) 12 h after transfection. In the above three experiments, cell samples were collected at 12, 24, and 36 h after CPV infection, and the expression of VP2 in F81 cells was detected by qPCR and western blot.

### 2.12 Data analysis and statistics

For statistical analysis of data, Student’s *t*-test was used to analyze significant differences between groups. When comparing multiple groups, one-way ANOVA and Dunnett’s multiple comparisons test were used. Three biological replicates were set up for all groups used for statistical analysis. The relevant statistical analysis was performed by GraphPad Prism (Version 9.0.0).

## 3 Results

### 3.1 Interaction between VP2 and CCT7 in yeast hybrid system

**FIGURE 1:**
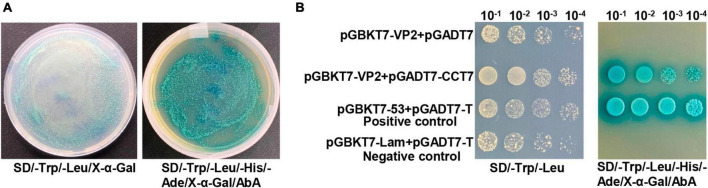
Yeast one-to-one assay to validate the interaction between CPV VP2 and CCT7. **(A)** The vectors pGADT7-CCT7 and pGBKT7-VP2 were transformed into Y2HGold competent yeast cells, and were plated onto SD/-Trp/-Leu/X-α-Gal and SD/-Trp/-Leu/-His/-Ade/X-α-Gal/ABA plates. **(B)** The prey (pGADT7-CCT7) and bait (pGBKT7-VP2) vectors were simultaneously transformed into yeast cells, while yeast strains harboring pGADT7-T/pGBKT7-Lam were used as negative control, and pGADT7-T/pGBKT7-53 were used as positive controls (yeast transformed with the β-galactosidase-positive plasmids will be blue), respectively. The obtained transformed samples were all diluted in a 10-fold series before plating.

VP2 protein is one of the important components in the viral capsid of CPV. To investigate the effect of VP2 on CPV replication, we used the yeast two-hybrid system and identified 28 potential VP2-interacting proteins (Supplementary Table 1), including the CCT7 subunit in the TRiC/CCT chaperonin complex (no other subunits in the TRiC/CCT chaperonin complex were detected). In order to confirm the interaction between CCT7 and VP2 in Y2HGold yeast strain, we used yeast two-hybrid test to carry out one-to-one verification. The results showed that yeast strains containing pGBKT7-VP2 and pGADT7-CCT7 plasmids could grow normally on SD/-Trp-Leu and SD/-Trp-Leu-His-Ade plates supplemented with X-α-gal and hydrolyze X-α-gal (Figure 1A), causing the yeast plaque to turn blue, which was consistent with the results of positive control yeast (containing pGBKT7- p53+pGADT7-T) (Figure 1B). This suggests a potential interaction between VP2 and CCT7 proteins.

### 3.2 Interaction of CCT7 with VP2 in F81 cells

**FIGURE 2:**
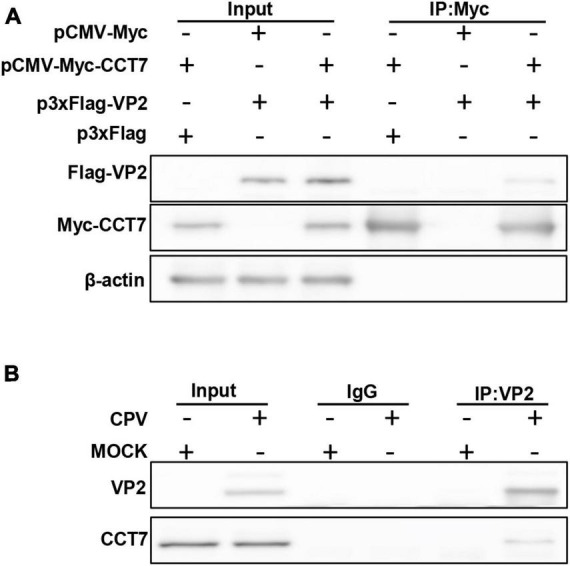
Validating the Interactions between CPV VP2 protein and CCT7 in F81 cells. **(A)** The interaction between VP2 and CCT7. pCMV-Myc-CCT7 and p3xFlag-VP2 plasmids were cotransfected in F81 cells, and the empty vector was used as a negative control. Immunoprecipitation was performed with anti-Myc magnetic beads and immunoblotting was performed using Myc-tagged and Flag-tagged antibodies. **(B)** Interaction of VP2 with endogenous CCT7 in F81 cells inoculated with CPV. F81 cells were infected with CPV (MOI = 0.1), and mock group were used as negative control. After 24 h of infection, cells were collected and immunoprecipitated with monoclonal antibodies against VP2. Immunoblotting was performed with antibodies against CCT7 and VP2. The IgG isotype control was employed at the same time.

To confirm the interaction between host protein CCT7 and CPV VP2 protein, we co-transfected Myc-VP2 and Flag-CCT7 into F81 cells to verify the interaction between CCT7 and VP2. When cell lysates expressing both CCT7 and VP2 were immunoprecipitated with anti-c-Myc magnetic beads, Flag-CCT7 was detected in the precipitates, indicating the interaction between CCT7 and VP2 (Figure 2A). To further determine the interaction of VP2 with CCT7, we infected F81 cells with CPV (MOI = 0.1) and collected the cells at 24 h post-infection. When cell lysates were precipitated with anti-VP2 antibody, endogenous CCT7 was detected in the precipitates, but no band was detected in the uninfected group (Figure 2B). The above results indicate that the VP2 protein interacts with the host protein CCT7.

### 3.3 Co-localization of CCT7 and VP2 in F81 cytoplasm

**FIGURE 3:**
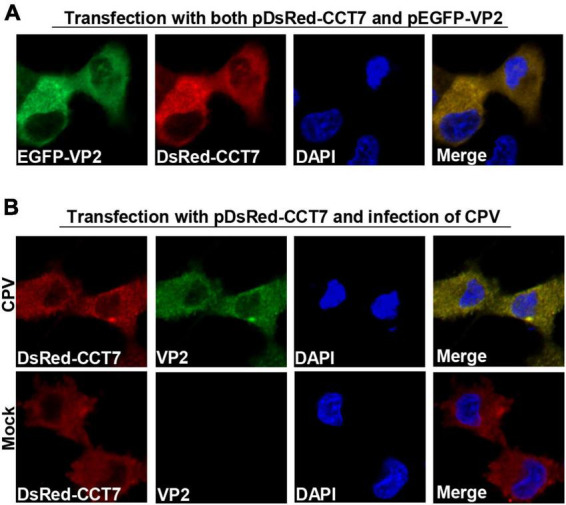
Colocalization of CPV VP2 with CCT7. **(A)** Colocalization of exogenous VP2 and CCT7. F81 cells were co-transfected with pDsRed-CCT7 and pEGFP-VP2 plasmids, 24 h later, the cells were fixed, the nuclei were stained with DAPI and observed by confocal microscope. Blue: nucleus, green: VP2, red: CCT7. **(B)** Colocalization of VP2 with exogenous CCT7 in F81 cells inoculated with CPV. F81 cells were transfected with pDsRed-CCT7 plasmid. At 12 h after transfection, 0.1 MOI dose of CPV was added to the cell. The cells were further cultured for 24 h. After fixation, the cells were incubated with VP2 antibodies for 1 h, followed by Goat anti-Mouse IgG (H+L) Secondary Antibody, FITC (green) for 1 h. The nuclei were stained with DAPI and observed by confocal microscope. Blue: nucleus, green: VP2, red: CCT7.

To determine the localization of CCT7 and VP2 in F81 cells, we co-transfected pDsRed-CCT7 and pEGFP-VP2 into F81 cells (Figure 3A). It was observed that VP2 and CCT7 were mainly co-localized in the cytoplasm of F81 cells. We further transfected pDsRed-CCT7 plasmid and then inoculated CPV (MOI = 0.1), similar results were also observed as above (Figure 3B). In addition, we infected F81 cells with CPV (MOI = 0.1), confocal microscopy observation showed that endogenous CCT7 and VP2 protein of CPV co-localized in the cytoplasm of the cells. However, due to the lack of a suitable CCT7 antibody that react with cat cells for confocal experiments, this data is provided as a Supplementary data to Figures 3A, B (Supplementary Figure 1).

### 3.4 Interaction region between VP2 and CCT7

**FIGURE 4:**
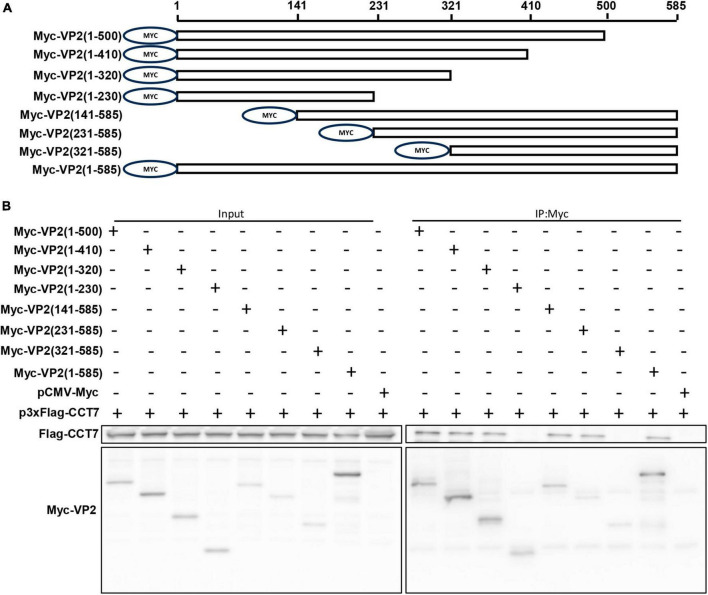
Identification of Interaction Regions between VP2 and CCT7. **(A)** Schematic diagram of VP2 truncated mutant construction. A series of VP2 truncated mutants were constructed and a Myc tag was added to the N-terminus of each truncated mutant. **(B)** Immunoprecipitation of VP2 and CCT7. F81 cells were transfected with wild-type or truncated mutant of VP2 plasmid and p3xFlag-CCT7 plasmid, respectively. At 24 hpi, immunoprecipitation was performed with Myc-tagged protein magnetic beads (coupled with Myc tags) and detected using Myc-tagged and Flag-tagged antibodies, respectively.

To identify the region of VP2 interaction with CCT7, we constructed a series of VP2 truncated mutants with c-Myc tag at the N-terminus of VP2 (Figure 4A). These truncated mutants were co-transfected with p3xFlag-CMV-CCT7 in F81 cells, respectively. Then the cells were collected at 24 hpi. The collected cell lysates were immunoprecipitated with anti-c-Myc magnetic beads, and the expression levels of CCT7 and VP2 deletion mutants were determined by immunoblotting with anti-c-Myc and anti-Flag antibodies, respectively (Figure 4B). It was found that the mutant with VP2 deletion of positions 231-320 failed to bind to CCT7, while the remaining truncated mutants were able to bind to CCT7. These results indicate that the amino acid region at positions 231–320 of VP2 protein plays an important role in the interaction with CCT7.

### 3.5 CCT7 affects the expression of VP2 protein

**FIGURE 5:**
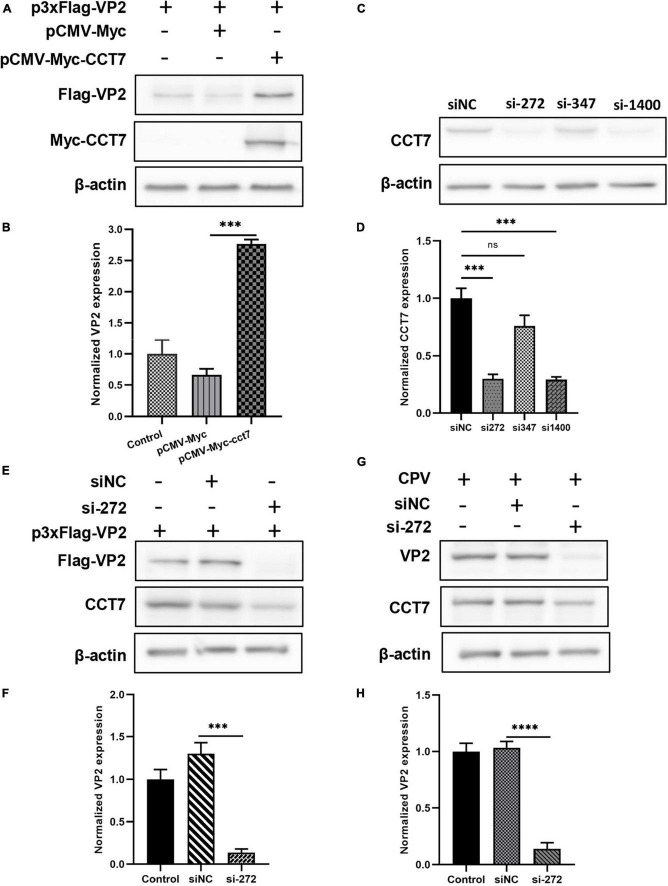
Effect of regulating CCT7 expression on VP2. **(A,B)** Effect of CCT7 overexpression on p3xFlag- VP2. F81 cells were co-transfected with pCMV-Myc, pCMV-Myc-CCT7, and p3xFlag-VP2 plasmids. After 24 h, cells were collected and analyzed for VP2 and CCT7 expression using western blot. The relative expression of VP2 in the samples was analyzed by Image J software. GAPDH was used as an internal control **(B)**. **(C,D)** CCT7 interference in F81 cells. The expression of CCT7 in F81 cells was knocked down by siRNA, and the cells were collected. The protein expression level of CCT7 was analyzed by western blot. The relative expression of VP2 in the samples was analyzed by Image J software. β-actin was used as an internal control **(D)**. **(E,F)** The effect of CCT7 interference on p3xFlag-VP2. F81 cells were transfected with p3xFlag-VP2 plasmid and siRNA-272 or control siNC. After 24 h, cells were collected and analyzed for VP2 and CCT7 expression using western blot. The relative expression of VP2 in the samples was analyzed by Image J software. GAPDH was used as an internal control **(F)**. **(G,H)** Effect of CCT7 interference on VP2 in CPV. F81 cells were transfected with siRNA-272 or control siNC, and then infected with CPV 12 h after the second round of transfection. After 24 h, cells were collected and analyzed for VP2 and CCT7 expression using western blot. The relative expression of VP2 in the samples was analyzed by Image J software. GAPDH was used as an internal control **(H)**. The significant differences on the statistical chart are represented by asterisks (***p < 0.005, ****p < 0.001, ns: no significant difference).

To verify the effect of CCT7 on VP2 protein expression, we co-transfected Myc-VP2 and Flag-CCT7 into F81 cells, and detected the effect of CCT7 on VP2 expression through western blot. The results showed that, compared with the Flag empty vector group, overexpression of CCT7 significantly increased the expression of VP2 protein (Figures 5A, B). In contrast, after using siRNA to interfere with the expression of endogenous CCT7 in host cell F81, we overexpressed Myc-VP2, and found that interfering with the expression of endogenous CCT7 in F81 cells significantly reduced the expression of VP2 compared with the siNC group (Figures 5C–F). In addition, after interfering with the expression of endogenous CCT7 in host cells F81, we inoculated CPV (MOI = 0.1), and found that compared with the siNC group, the expression of VP2 in the CCT7 knockdown group decreased, and the copy number of CPV also decreased (Figures 5G, H). The results indicate that CCT7 can regulate the expression of VP2, which in turn affects the proliferation of CPV.

### 3.6 Cycloheximide (CHX) chase experiment to verify the improvement of VP2 stability by CCT7

**FIGURE 6:**
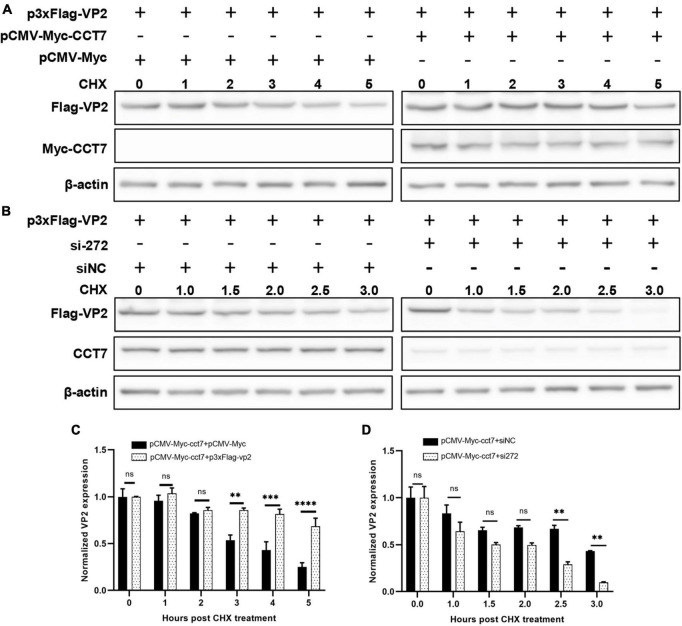
CCT7 was involved in the stabilization of VP2 in F81 cells. **(A,C)** F81 cells were transfected with pCMV-Myc-CCT7 and p3xFlag-VP2 plasmids. pCMV-Myc was used as an empty vector control, and CHX was added after 20 h. Cells were collected at 0, 1, 2, 3, 4, and 5 h after the addition of CHX. Protein expression level of VP2 and CCT7 were analyzed by western blot. The relative expression of VP2 in the samples was analyzed by Image J software. β-actin was used as an internal control (C). **(B,D)** F81 cells were transfected with siRNA or control siNC, respectively, and then infected with CPV. The incubation was continued for 20 h and then CHX was added, and cells were collected at 0, 1, 1.5, 2.0, 2.5, and 3.5 h after the addition of CHX. Protein expression levels of VP2 and CCT7 in the samples were analyzed by western blot and the relative expression of VP2 in the sample was analyzed by Image J software. β-actin was used as internal control **(D)**. The significant differences on the statistical chart are represented by asterisks (**p < 0.01, ***p < 0.005, , ****p < 0.001, ns: no significant difference).

To further determine the effect of CCT7 on VP2, we co-transfected F81 cells with p3xFlag-CMV-14-CCT7 and pCMV- Myc-VP2 as well as a control empty vector, and then treated the cells with cycloheximide (CHX, a protein synthesis inhibitor) (50 μg/mL), and then the expression levels of VP2 and CCT7 at different CHX addition times (0, 1, 2, 3, 4, and 5 h) were detected by western blot assay. The results showed that although the expression of VP2 in the overexpressing CCT7 group also gradually decreased with the longer time interval post CHX treatment, the decrease of VP2 was significantly less than that in the group without overexpressing CCT7 (Figures 6A, C). In contrast, cells in which CCT7 expression was knocked down and transfected with the pCMV-Myc-VP2 plasmid were also treated with the protein synthesis inhibitor CHX (50 μg/mL), and the cells were collected at different time points (0, 1, 1.5, 2.0, 2.5, and 3.5 h) after CHX treatment. Detection of VP2 and CCT7 expression levels showed that after knocking down the expression level of CCT7 in host cells, the decrease of VP2 expression was more obvious than that in the siNC group (Figures 6B, D). The above results indicate that host protein CCT7 affects the stability of CPV VP2 protein.

### 3.7 The effect of CCT7 on the replication of CPV virus

**FIGURE 7:**
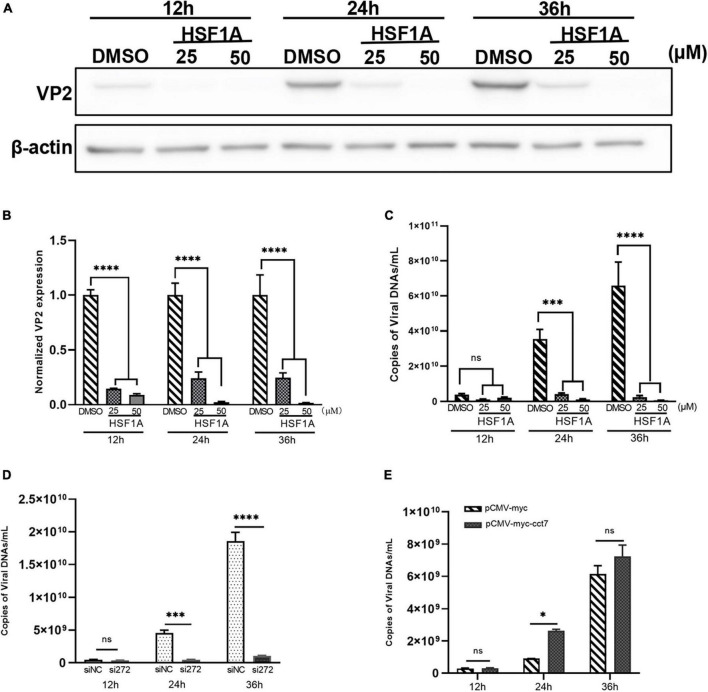
Effect of regulating CCT7 on CPV replication. **(A–C)** The effect TRiC/CCT inhibitor HSF1A on CPV replication. F81 cells infected by CPV were treated with different concentrations of HSF1A, and the cells were collected at 12, 24 and 36 h after CPV infection. The protein expression level of CCT7 was analyzed by western blot **(A)**. The relative expression of VP2 in the samples was analyzed by Image J software. β-actin was used as an internal control **(B)**. The virus copy number of CPV was detected by qPCR **(C)**. **(D)** The effect of knocking down CCT7 on CPV replication. F81 cells were transfected with siRNA-272 or control siNC, and then infected with CPV. Cells were collected at 12, 24 and 36 h after CPV infection. The virus copy number of CPV was detected by qPCR. **(E)** The effect of overexpression of CCT7 on CPV replication. F81 cells were transfected with pCMV-Myc and pCMV-Myc-CCT7 plasmids, respectively, and infected with CPV 12 h after transfection. Cells were collected at 12, 24 and 36 h after CPV infection. The virus copy number of CPV was detected by qPCR. The significant differences on the statistical chart are represented by asterisks (*p < 0.05, ***p < 0.005, ****p < 0.001, ns: no significant difference).

To further study whether CCT7 affects CPV replication in F81 cells, a TRiC/CCT inhibitor HSF1A (inhibits TRiC activity by binding to TRiC subunits) was used to inhibit the expression of TRiC/CCT in F81 cells infected with CPV. qPCR and western blot assays both showed that HSF1A treatment resulted in a significant decrease in the copy number or expression of CPV VP2 compared to the 0.1% DMSO control group (Figures 7A–C). Furthermore, we used siRNA and overexpression of exogenous CCT7 to regulate CCT7 expression in F81 cells. The results of qPCR assay showed that after knocking down the expression of CCT7, the copy number of CPV VP2 was significantly reduced compared with the control group (Figure 7D). In contrast, the copy number of CPV VP2 was significantly increased after increasing the expression of CCT7 (Figure 7E). The above results suggest that modulation of CCT7 expression can affect CPV replication in F81 cells.

## 4 Discussion

Viruses can control their infection and replication by regulating the G1/S and G2/M boundaries (Miranda and Thompson, 2016; Qi et al., 2020). At present, CPV-related pathogenic molecular mechanisms are mainly focused on the effects of NS1 protein on apoptosis, cell cycle, and related signaling pathways (Gupta et al., 2016; Lin et al., 2019; Dai et al., 2020). In addition, studies have shown that the cellular DNA damage response triggered by the invading single-stranded parvovirus can induce cell cycle arrest, thereby creating a favorable environment for viral replication (Chen and Qiu, 2010; Lou et al., 2012; Xu et al., 2019; Zhang et al., 2019; Dai et al., 2020). While as an important component of the viral capsid, the CPV VP2 protein not only determines the host range of the virus (Hueffer and Parrish, 2003), but also serves as a key protein in inducing the host immune defense mechanism (Sarabandi and Pourtaghi, 2023). It follows that VP2 protein plays a crucial role in the biological functions related to virus-host interactions. However, the role of VP2 in mediating viral infection remains poorly known. To this end, we used the yeast two-hybrid system to identify the host cell protein CCT7 that interacts with CPV VP2, and further elucidate the role of CCT7 in virus infection.

Our research data indicate that the interaction region between CCT7 and VP2 is located in the (amino acids 231–320) region of VP2 (Figure 3), and VP2 loses its ability to bind to CCT7 when this region is missing. Previous studies showed that the 300 and 301 amino acids at loop3 (amino acids 284–310) position in the CPV genome are two major neutralizing antigen sites, which are located on the shoulder of the VP2 protein (Tsao et al., 1991; Strassheim et al., 1994; Agbandje-McKenna et al., 1998). Furthermore, the shoulder of the VP2 protein (amino acids 295–306) plays a crucial role in the interaction between the virus and canine TfR, which is involved in the change of protein conformation (Lee et al., 2019). It has also been reported that amino acids 297, 300, 301, and 305 of CPV VP2 protein were conserved in different vaccine strains, and these sites affect the three-dimensional structure of the protein (Silva et al., 2022). The interaction region identified in this study (amino acids 231–320) contains the above regions and sites, once again demonstrating the importance of this region in CPV infection and replication.

Wang et al. (2022) found in their study of glioblastoma that the TRiC/CCT chaperone protein CCT4 can activate the mTOR signaling pathway by protecting mLST8 protein from lysosomal degradation caused by misfolding, thus promoting the growth of glioblastoma. Liu et al. (2019) found that the expression of TRiC/CCT subunit 3 (CCT3) was increased in liver cancer, and further studies showed that CCT3 could enhance the protein stability of yes-associated protein (YAP) and transcription factor CP2 (TFCP2) by reducing their ubiquitination. It has also been shown that TRiC/CCT chaperone proteins (CCT2 and CCT5) can control avian reovirus (ARV) replication by protecting the ARV outer capsid protein σC and inner core protein σA, as well as the non-structural protein σNS, from ubiquitin-proteasome degradation (Huang et al., 2022). These studies indicate that TRiC/CCT chaperonin complex affects the stability of interacting proteins. In this study, we treated CPV-infected F81 cells with protease inhibitor (CHX), and found that CCT could stabilize VP2 expression, and the presence of CCT slowed down the decreasing trend of VP2 protein.

Cell cycle arrest and apoptosis and related pathways play important roles in CPV replication. For example, CPV can trigger host G1/S cell cycle arrest via the EGFR (Y1086)/p27 and EGFR (Y1068)/STAT3/cyclin D1 axis (Dai et al., 2020), and in the late stage of viral infection, nuclear assembly of the progeny capsids is accompanied by virus-induced cell cycle triggering host cell G2/M phase arrest (Adeyemi et al., 2010; Ruiz et al., 2011; Mattola et al., 2022). Studies have shown that the eukaryotic chaperone T-complex protein-1 (TCP-1) ring complex (TRiC) plays an important role in the folding of many apoptosis and cell cycle-related proteins. TRiC/CCT chaperone protein subunits, CCT2 and CCT5, can stabilize CDC20 and mediate Muscovy duck reovirus (MDRV) p10.8 and σ C-protein induced cell cycle arrest and apoptosis, and CDC20 depletion reversed p10.8 and σC-mediated CDK4 degradation and p10.8-induced cell apoptosis (Wang et al., 2019). In addition, Liao et al. (2021) showed in a research on tumor progression in colorectal cancer (CRC) that there is an endogenous interaction between the TRiC/CCT chaperone protein subunit CCT8 and p53 in CRC cells. CCT8 promotes cell cycle evolution of CRC by inhibiting the entry of p53 into the nucleus (Liao et al., 2021). However, whether the regulation of CPV replication by the TRiC/CCT chaperone subunit CCT7 is related to the cell cycle still needs further study.

## 5 Conclusion

In this study, we found that CCT7 interacts with VP2 and was co-localized in the cytoplasm. The interaction region of VP2 with CCT7 was located between amino acids 231 and 320. CHX chase experiments showed that CCT7 can improve the stability of VP2 protein. In addition, Regulating of CCT7 expression affects CPV replication. Knockdown of CCT7 expression by RNA interference (RNAi) or HSF1A inhibitor decreased VP2 expression and inhibited CPV replication, while overexpression of CCT7 in host cells promoted CPV replication. The study reveals the role of VP2 interacting protein CCT7 in the replication process of CPV, which could provide a potential target for the prevention and control of CPV.

## Data availability statement

The original contributions presented in this study are included in this article/Supplementary material, further inquiries can be directed to the corresponding author.

## Ethics statement

Ethical approval was not required for the studies on animals in accordance with the local legislation and institutional requirements because only commercially available established cell lines were used.

## Author contributions

XS: Conceptualization, Formal analysis, Methodology, Software, Writing – original draft. HZ: Conceptualization, Formal analysis, Methodology, Software, Writing – original draft. FX: Conceptualization, Formal analysis, Software, Writing – original draft. JZ: Formal analysis, Methodology, Software, Writing – review & editing. BX: Formal analysis, Methodology, Writing – review & editing. QQ: Formal analysis, Methodology, Writing – review & editing. LL: Formal analysis, Methodology, Writing – review & editing. BY: Conceptualization, Software, Writing – original draft.
