# Supplementary material for: Chaperonin TRiC/CCT subunit CCT7 is involved in the replication of canine parvovirus in F81 cells
Source: Front Microbiol. 2024 Feb 7;15:1346894. doi: 10.3389/fmicb.2024.1346894 (PMC10879588; doi:10.3389/fmicb.2024.1346894)
Supplement: Supplementary file 1 [file Data_Sheet_1.docx]

Supplementary Material

# Supplementary Figure and Table

## Supplementary Figure


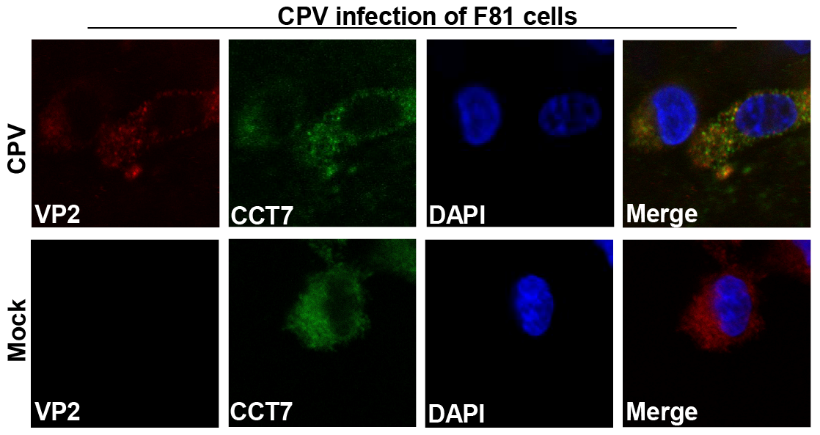


**Supplementary Figure 1.** Colocalization of endogenous CCT7 and VP2. After 24 h of infection with CPV, F81 cells were fixed and incubated with a mixture of anti-CCT7 and anti-VP2 antibodies for 1 h. Goat anti-Mouse IgG (H+L) Cross-Adsorbed Secondary Antibody, TRITC (red) and Goat anti-Rabbit IgG (H+L) Secondary Antibody, DyLight™ 488 (green) mixed secondary antibody were incubated for 1h. Cell nuclei were stained with DAPI and observed by confocal microscope. Blue: nuclei, green: CCT7, red: VP2.

## Supplementary Table

**Supplementary Table 1. List of potential interacting proteins of VP2.**

| Clones | Genbank | Gene Name | Description |
| --- | --- | --- | --- |
| VP2-clone-01 | XM_023243760.2 | PIMREG | protein PIMREG isoform X2 |
| VP2-clone-02 | XM_003997930.4 | RBM42 | RNA-binding protein 42 isoform X1 |
| VP2-clone-03 | XM_003981464.5 | BRIX1 | ribosome biogenesis protein BRX1 homolog |
| VP2-clone-04 | XM_003981464.5 | BRIX1 | ribosome biogenesis protein BRX1 homolog |
| VP2-clone-05 | XM_003982375.5 | PDHB | pyruvate dehydrogenase E1 component subunit beta, mitochondrial |
| VP2-clone-06 | XM_045054907.1 | WDR35 | WD repeat domain 35, transcript variant X3 |
| VP2-clone-07 | XM_023243853.2 | GAS7 | growth arrest-specific protein 7 isoform X1 |
| VP2-clone-08 | XM_003988644.6 | LOC101096402 | tubulin alpha-1B chain |
| VP2-clone-09 | XM_023240590.2 | VDAC2 | voltage-dependent anion-selective channel protein 2 |
| VP2-clone-10 | XM_023251253.2 | TOP1 | DNA topoisomerase I |
| VP2-clone-11 | XM_011281113.3 | PCBP1 | poly(rC)-binding protein 1 |
| VP2-clone-12 | XM_011285657.4 | PAX3 | paired box 3 |
| VP2-clone-13 | XM_003998635.4 | FUS | RNA-binding protein FUS isoform X1 |
| VP2-clone-14 | XM_003987616.5 | POLE2 | DNA polymerase epsilon subunit 2 isoform X1 |
| VP2-clone-15 | XM_006941918.5 | LMTK2 | lemur tyrosine kinase 2, transcript variant X1 |
| VP2-clone-16 | XM_011283973.4 | SMUG1 | single-strand-selective monofunctional uracil-DNA glycosylase 1, transcript variant X1 |
| VP2-clone-17 | XM_003990961.4 | COL3A1 | collagen alpha-1(III) chain |
| VP2-clone-18 | XM_023258405.2 | NR2F1 | COUP transcription factor 1 isoform X1 |
| VP2-clone-19 | XM_003984121.5 | CCT7 | T-complex protein 1 subunit eta |
| VP2-clone-20 | XM_003990621.5 | PSMB4 | proteasome subunit beta type-4 |
| VP2-clone-21 | XM_006940882.5 | CNOT3 | CCR4-NOT transcription complex subunit 3 isoform X1" |
| VP2-clone-22 | XM_023250126.2 | LOC101099622 | histone H2A.V isoform X2 |
| VP2-clone-23 | XM_023256248.2 | CD63 | CD63 antigen isoform X1 |
| VP2-clone-24 | XM_023245980.2 | N4BP1 | NEDD4-binding protein 1 isoform X1 |
| VP2-clone-25 | XM_045053984.1 | FHL2 | four and a half LIM domains protein 2 |
| VP2-clone-26 | XM_011291501.4 | PUF60 | poly(U)-binding-splicing factor PUF60 isoform X1" |
| VP2-clone-27 | XM_003994318.6 | HIF1AN | hypoxia-inducible factor 1-alpha inhibitor |
| VP2-clone-28 | XM_011287727.4 | EWSR1 | RNA-binding protein EWS isoform X4 |
| VP2-clone-29 | XM_023252110.2 | PDIA6 | protein disulfide-isomerase A6 |
