# Supplementary material for: Chaperonin TRiC/CCT subunit CCT7 is involved in the replication of canine parvovirus in F81 cells
Source: Front Microbiol. 2024 Feb 7;15:1346894. doi: 10.3389/fmicb.2024.1346894 (PMC10879588; doi:10.3389/fmicb.2024.1346894)

FIGURE 1A

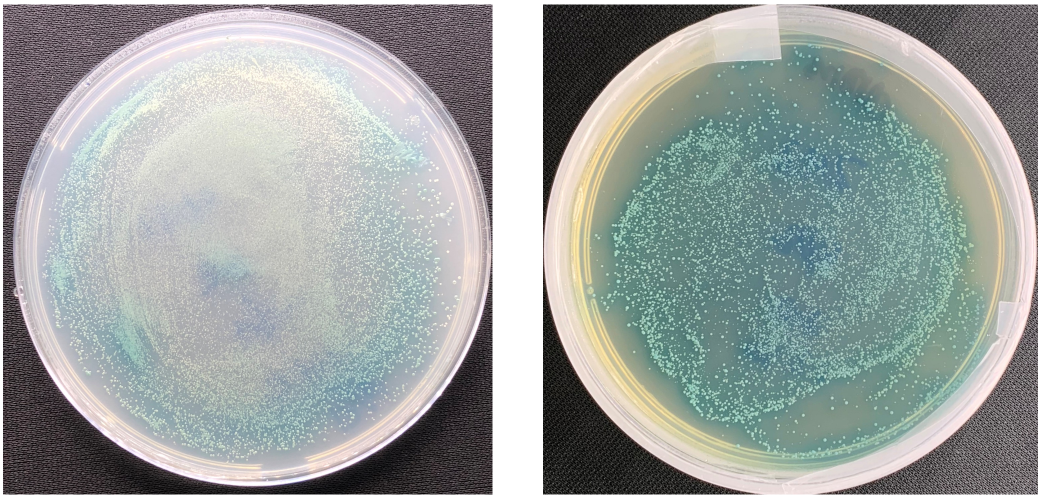

SD/-Trp/-Leu/X-α-Gal   SD/-Trp/-Leu/-His/-Ade/X-α-Gal/AbA

FIGURE 1B

10^{-1} 10^{-2} 10^{-3} 10^{-4} 10^{-1} 10^{-2} 10^{-3} 10^{-4}

pGBKT7-VP2+pGADT7

pGBKT7-VP2+pGADT7-CCT7

pGBKT7-53+pGADT7-T
Positive control

pGBKT7-Lam+pGADT7-T
Negative control

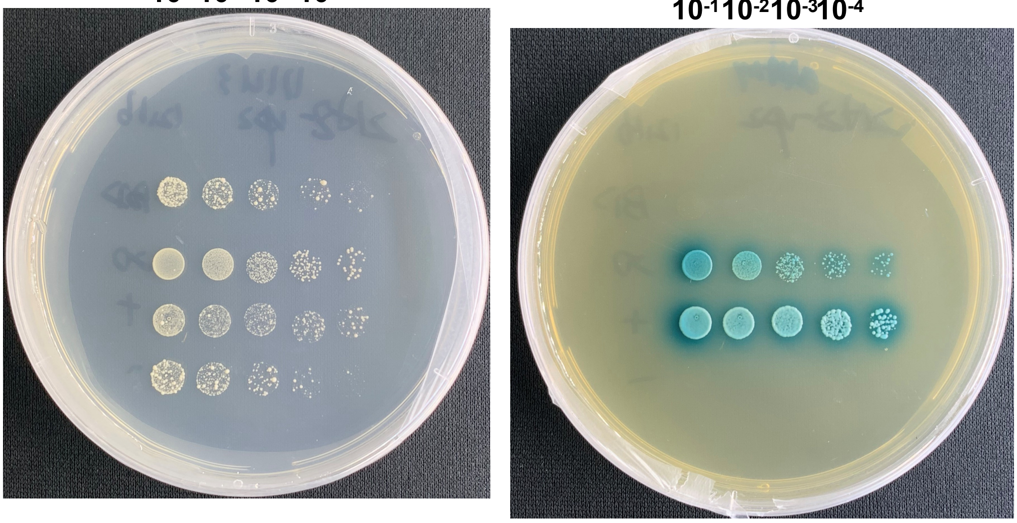

SD/-Trp/-Leu   SD/-Trp/-Leu/-His/-Ade/X-α-Gal/AbA

FIGURE 2A

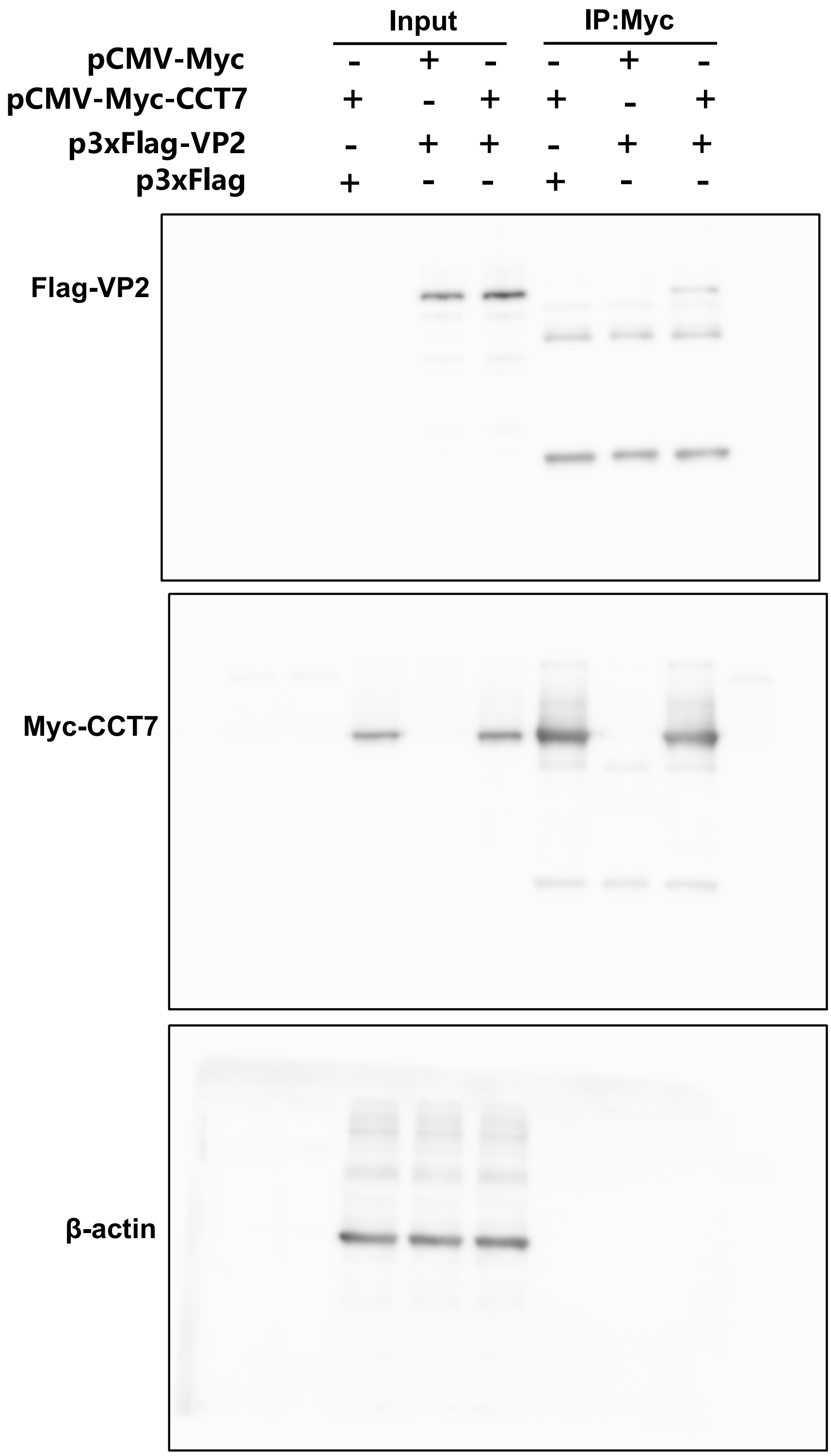
Input
IP:Myc
pCMV-Myc - + - - + -
pCMV-Myc-CCT7 + - + + - +
p3xFlag-VP2 - + + - + +
p3xFlag + - - + - -
Flag-VP2
Myc-CCT7
β-actin

FIGURE 2B

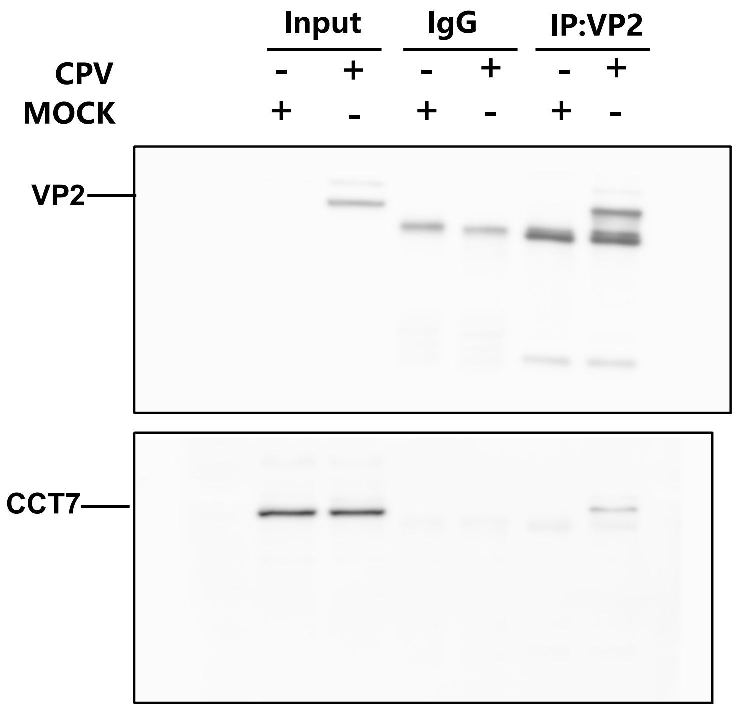

FIGURE 4B

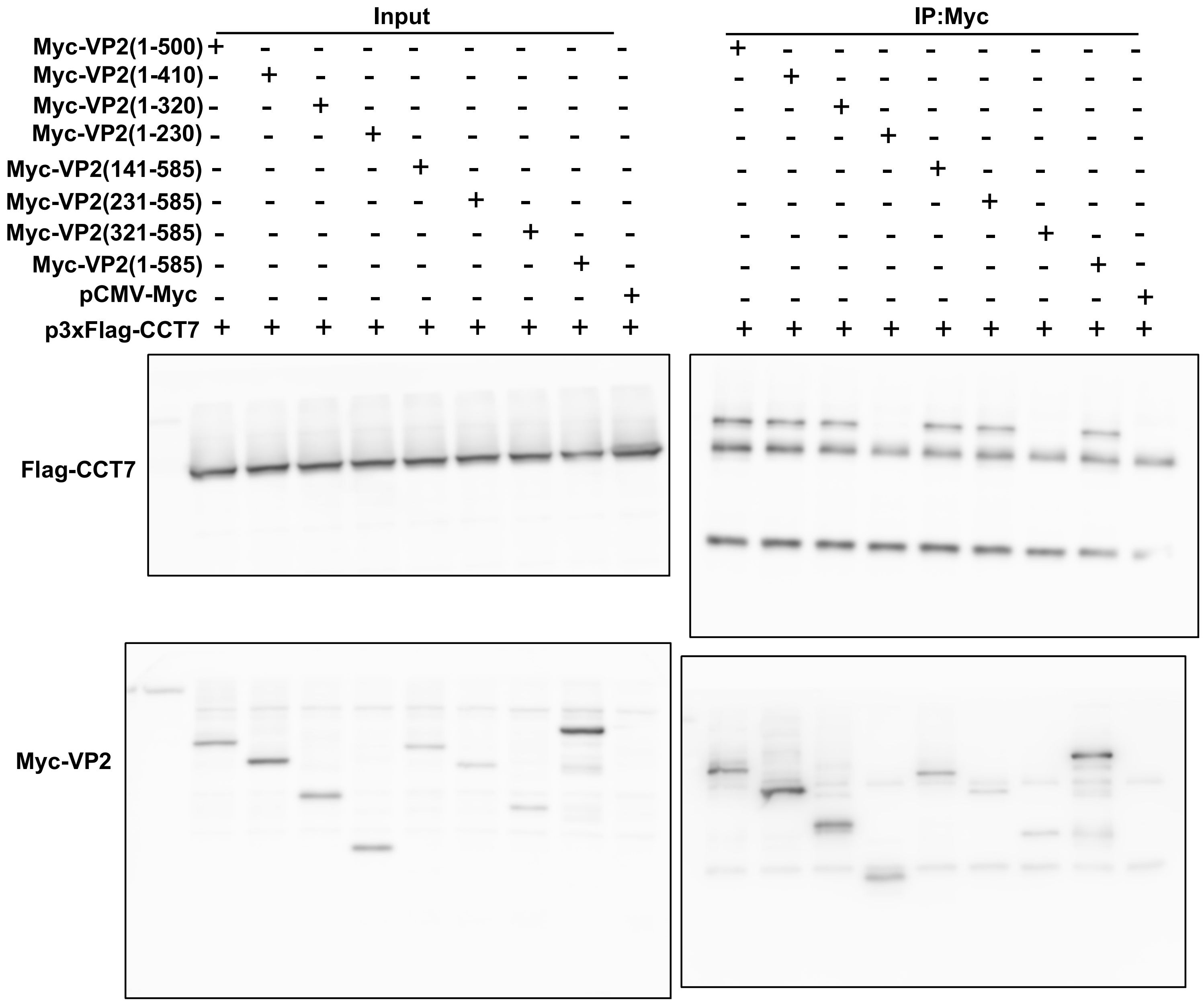
Input
IP:Myc
Myc-VP2(1-500)
Myc-VP2(1-410)
Myc-VP2(1-320)
Myc-VP2(1-230)
Myc-VP2(141-585)
Myc-VP2(231-585)
Myc-VP2(321-585)
Myc-VP2(1-585)
pCMV-Myc
p3xFlag-CCT7
Flag-CCT7
Myc-VP2

FIGURE 5A

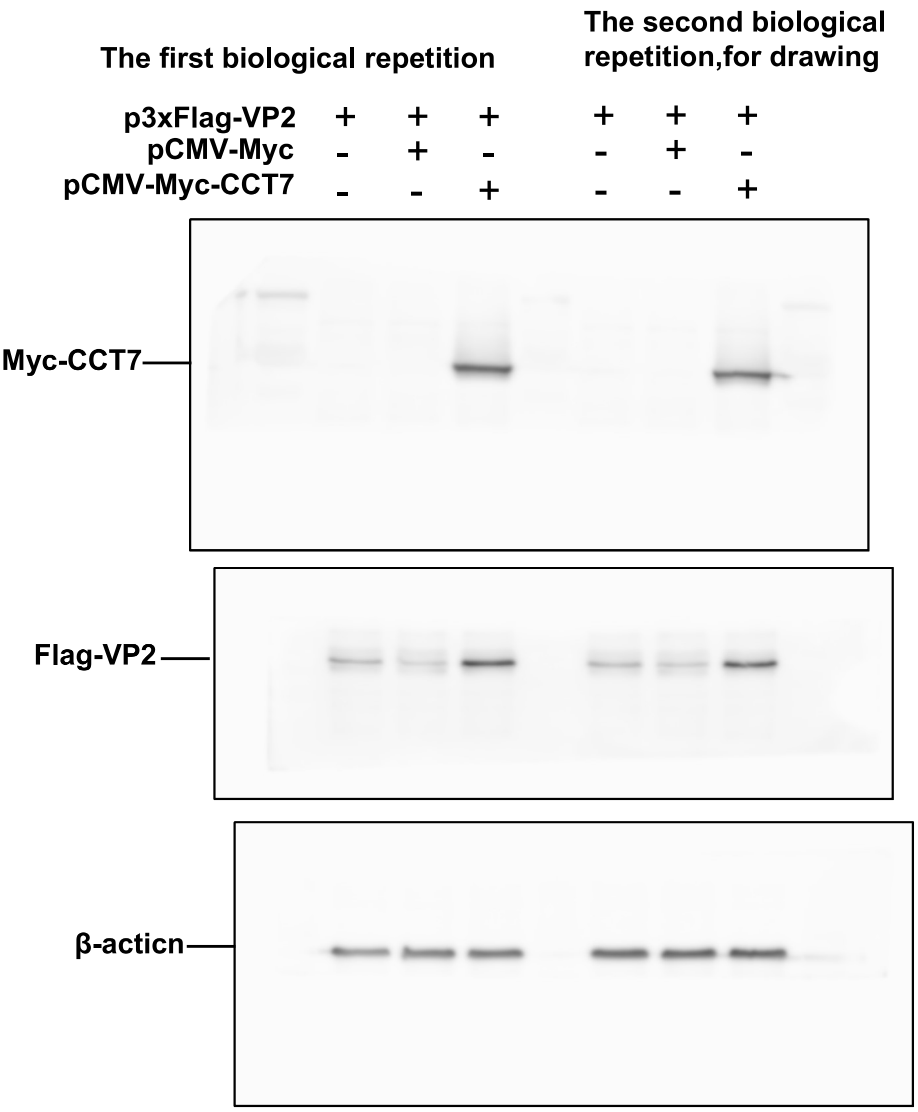
The first biological repetition
The second biological repetition,for drawing
p3xFlag-VP2 + + + + + +
pCMV-Myc - + - - + -
pCMV-Myc-CCT7 - - + - - +
Myc-CCT7
Flag-VP2
β-acticn

FIGURE 5A

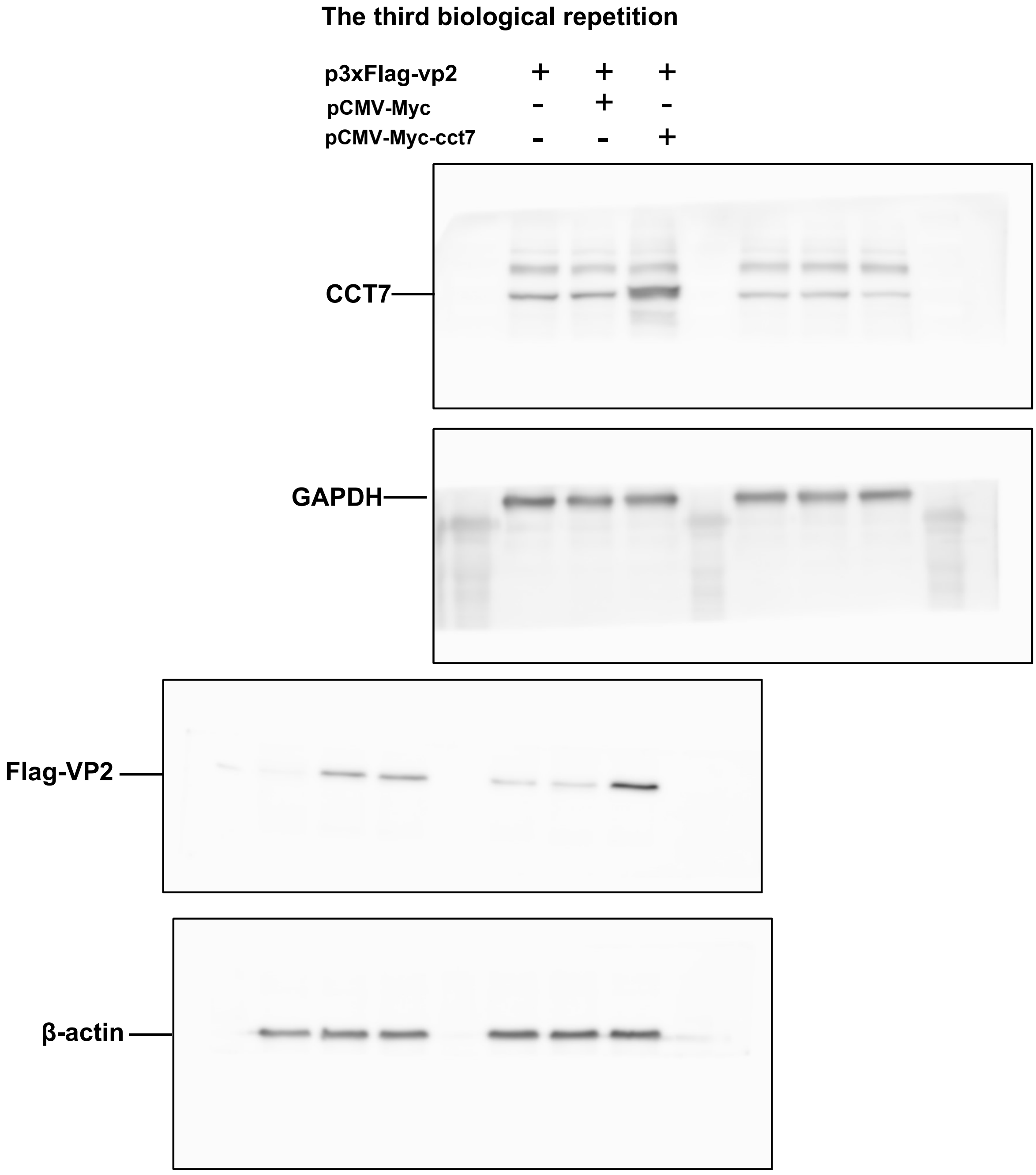

The third biological repetition
p3xFlag-vp2 + + +
pCMV-Myc - + -
pCMV-Myc-cct7 - - +
CCT7
GAPDH
Flag-VP2
β-actin

FIGURE 5C

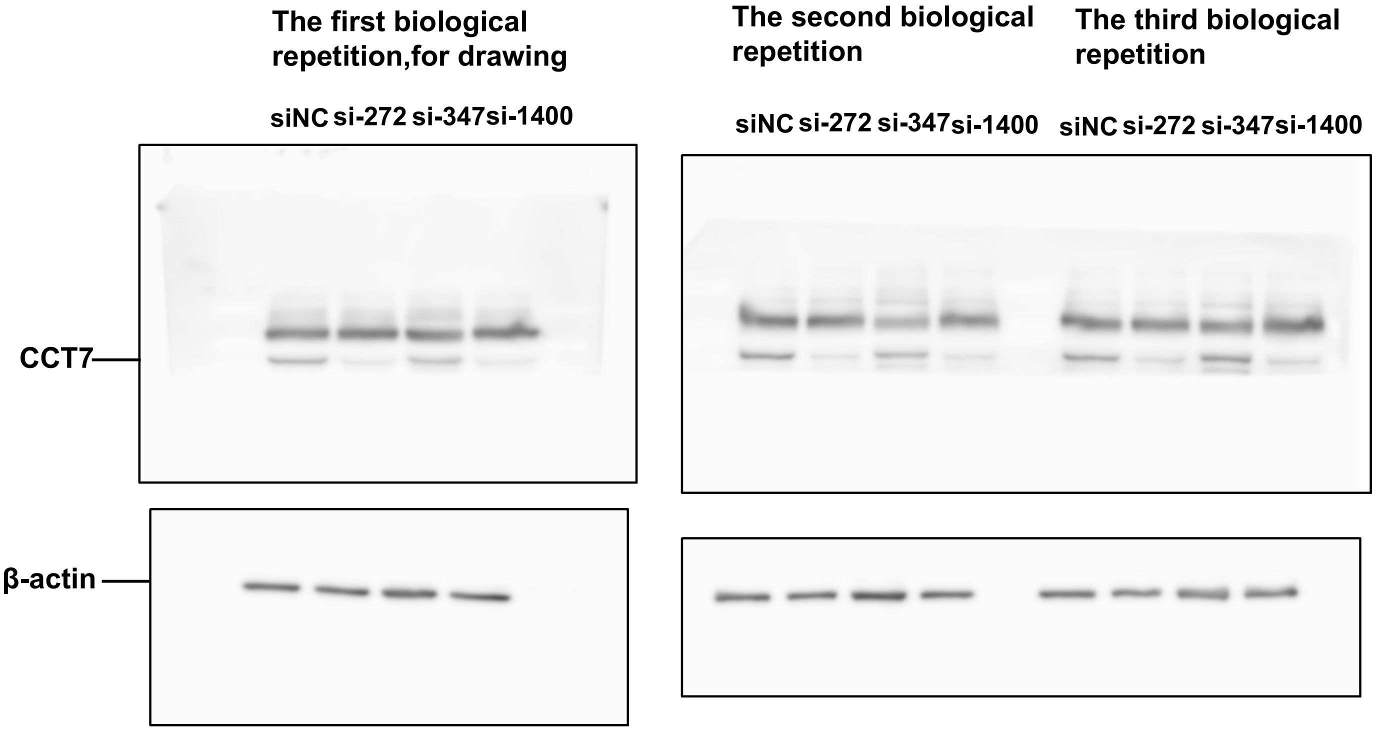

FIGURE 5E

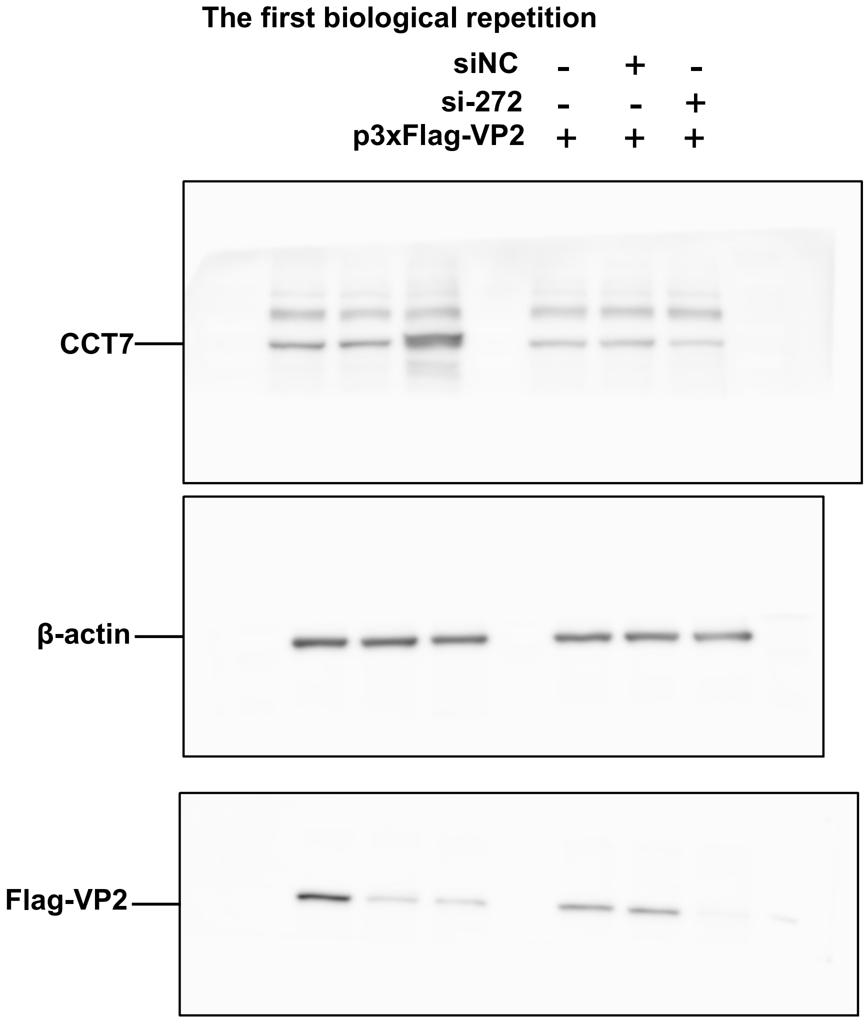

FIGURE 5E

The second biological repetition

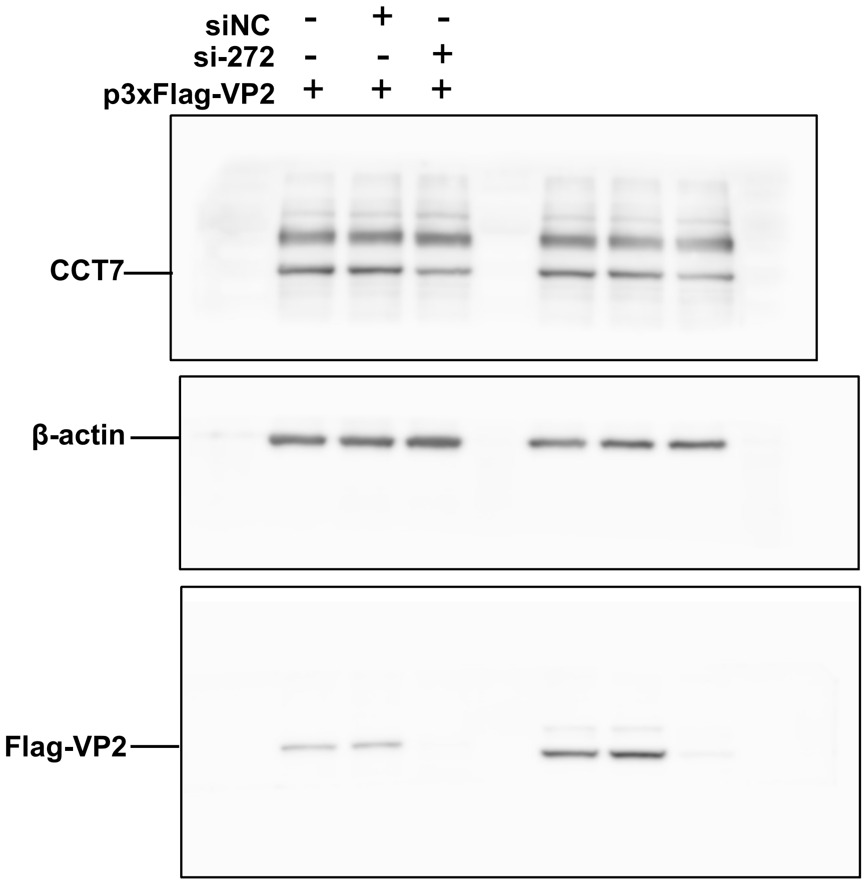

The third biological repetition,for drawing

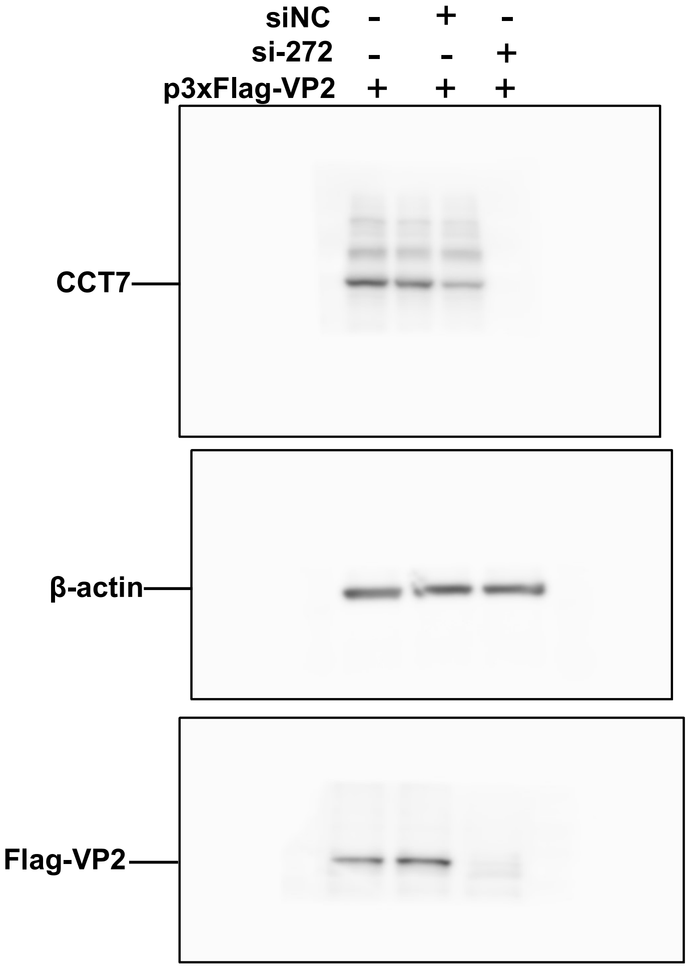

FIGURE 5G

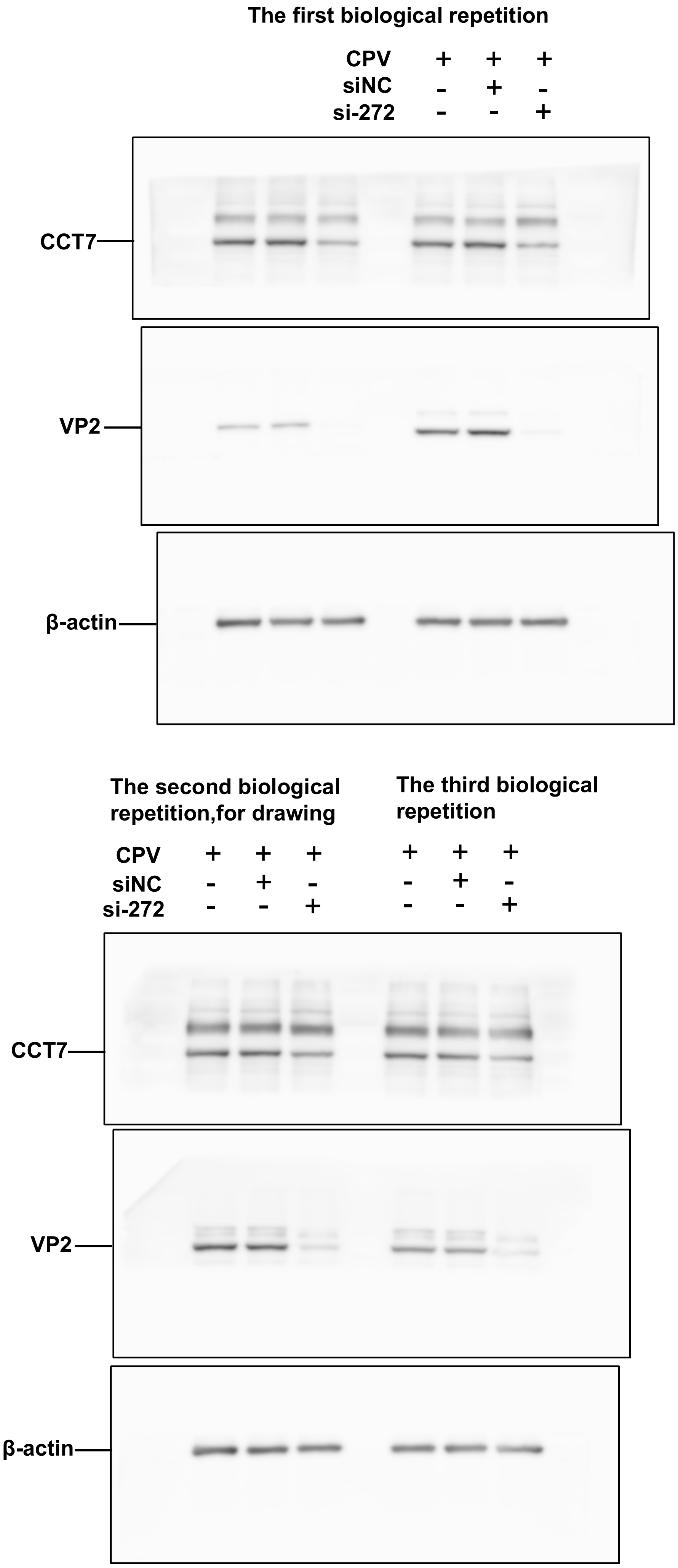
The first biological repetition
CPV + + +
siNC - + -
si-272 - - +
CCT7
VP2
β-actin
The second biological repetition,for drawing
The third biological repetition
CPV + + + + + +
siNC - + - - + -
si-272 - - + - - +
CCT7
VP2
β-actin

FIGURE 6A

The first biological repetition,for drawing

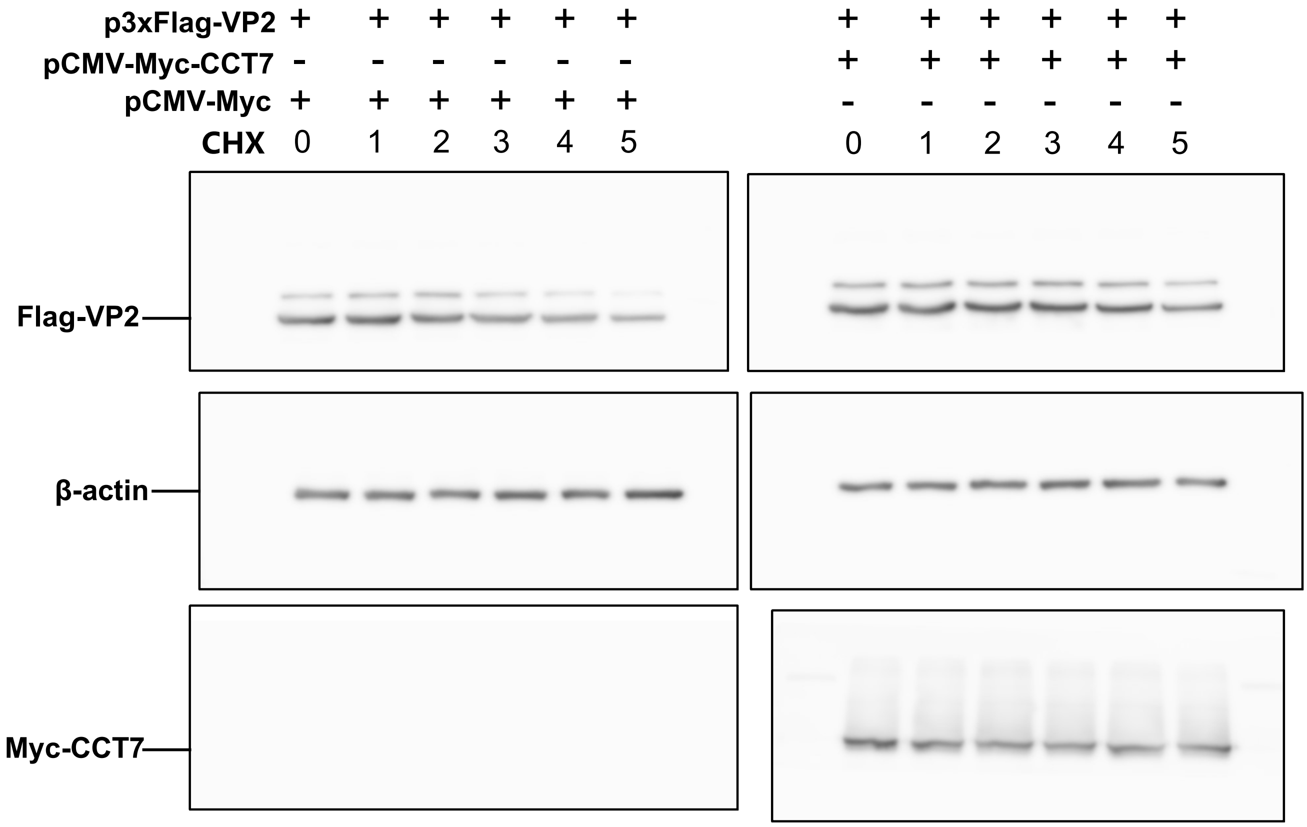

The second biological repetition

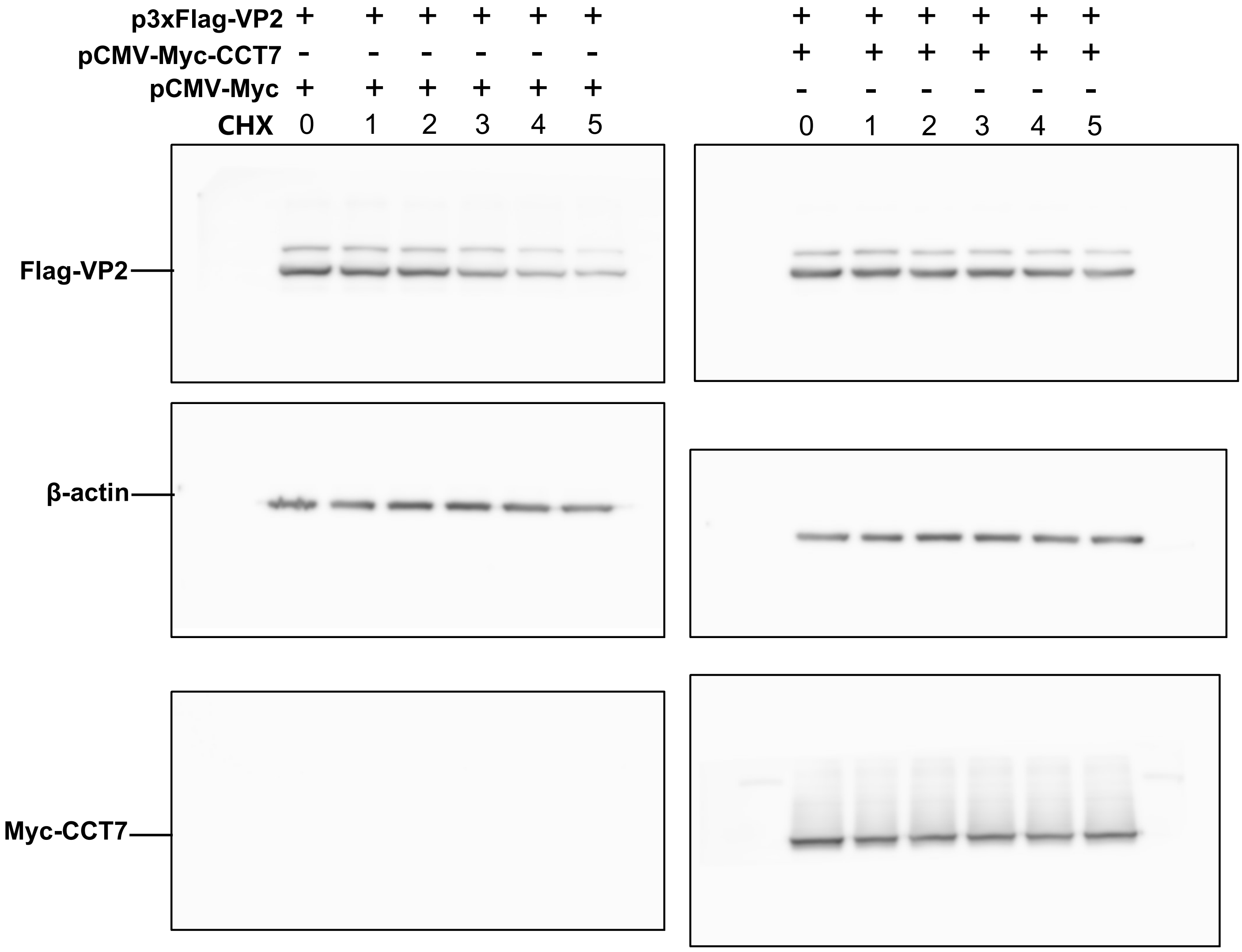

The third biological repetition

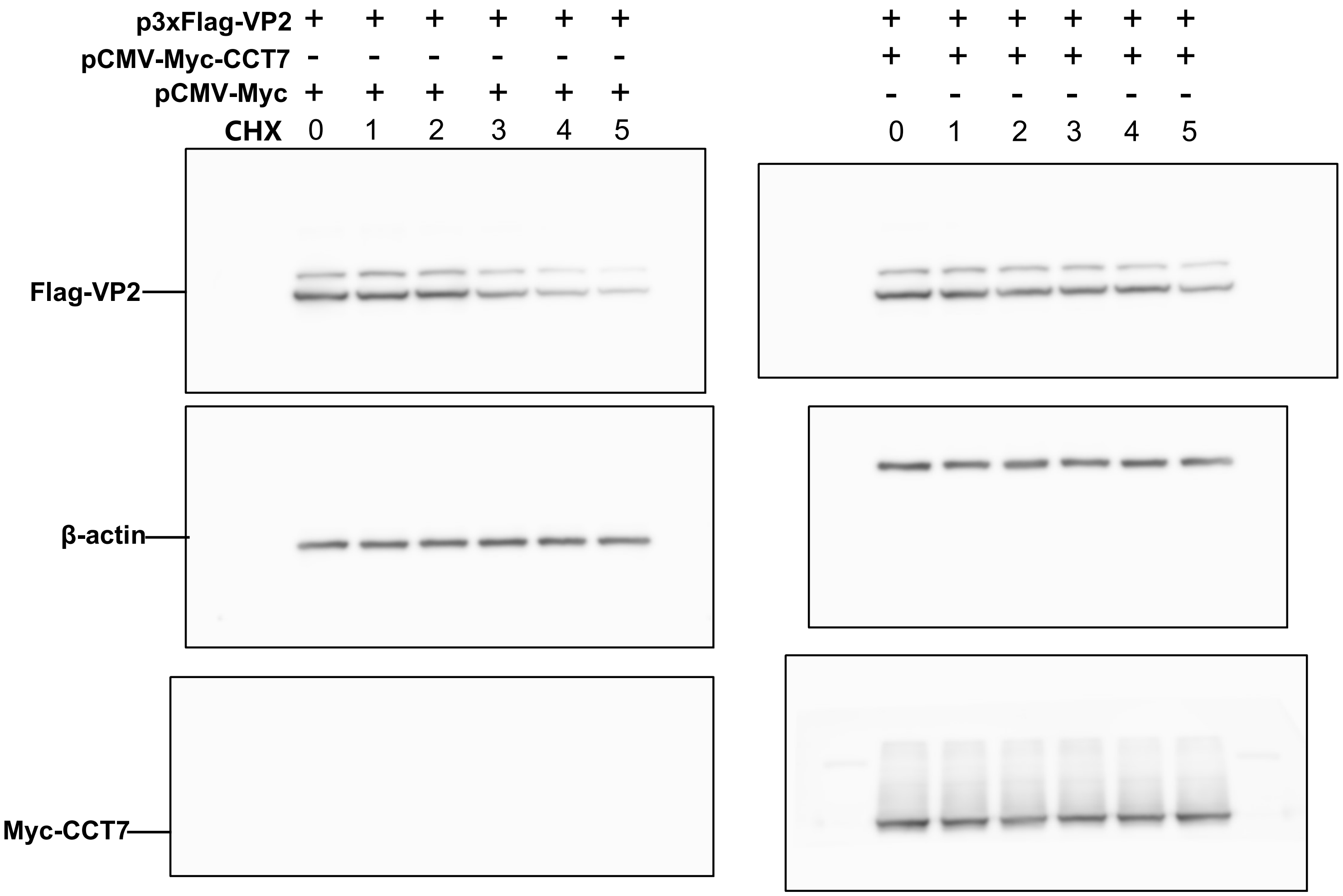

FIGURE 6B

The first biological repetition

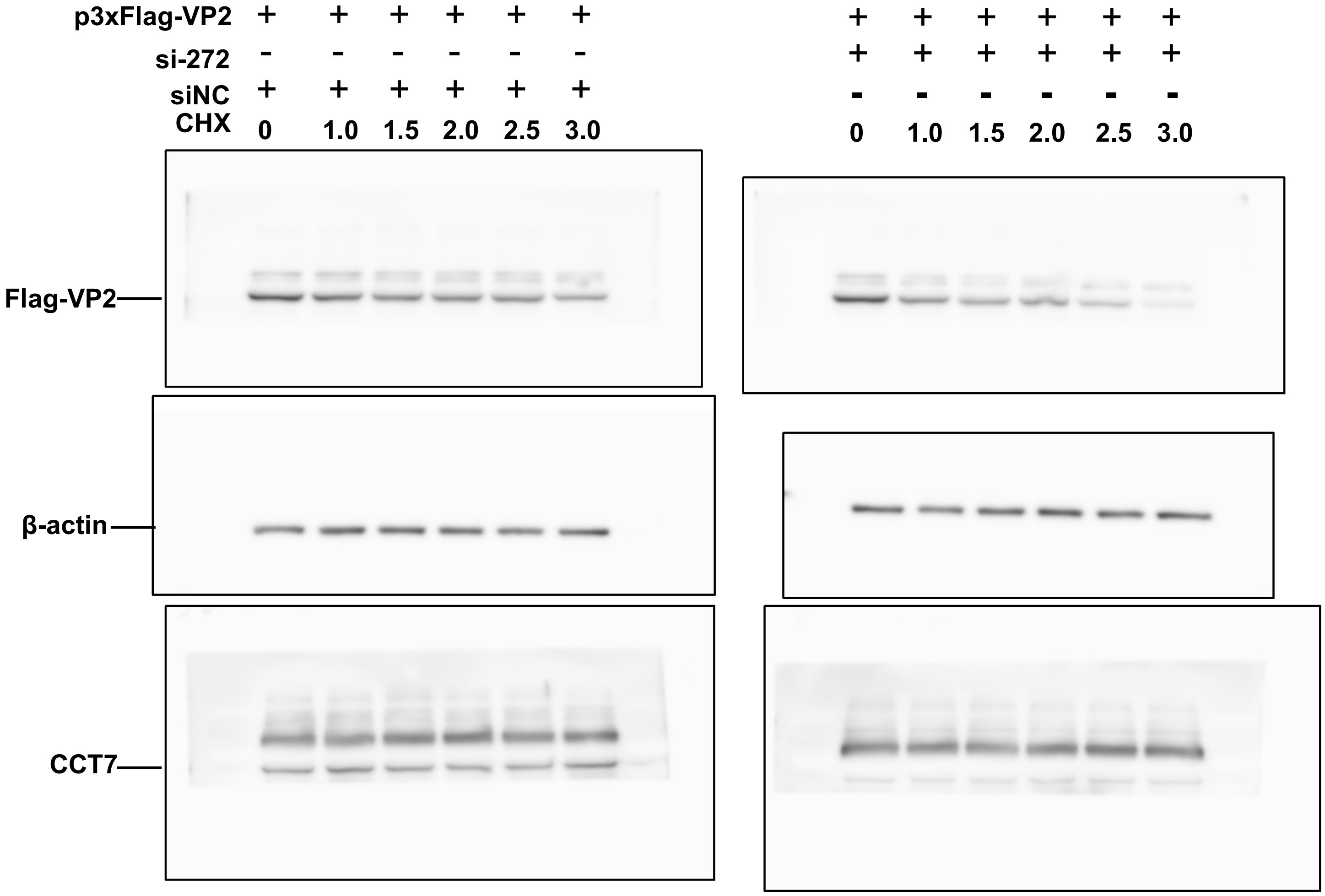

The second biological repetition,for drawing

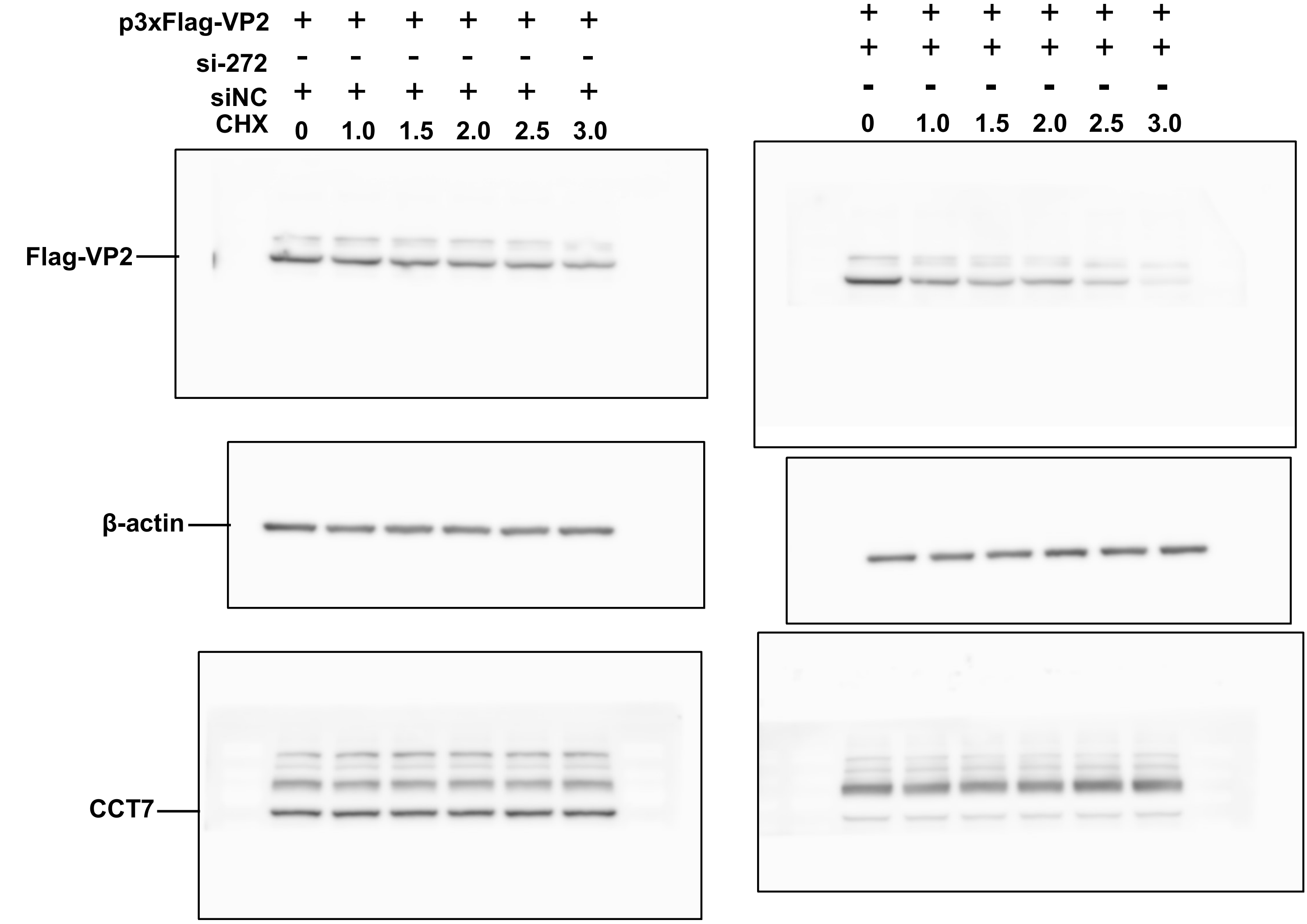

The third biological repetition

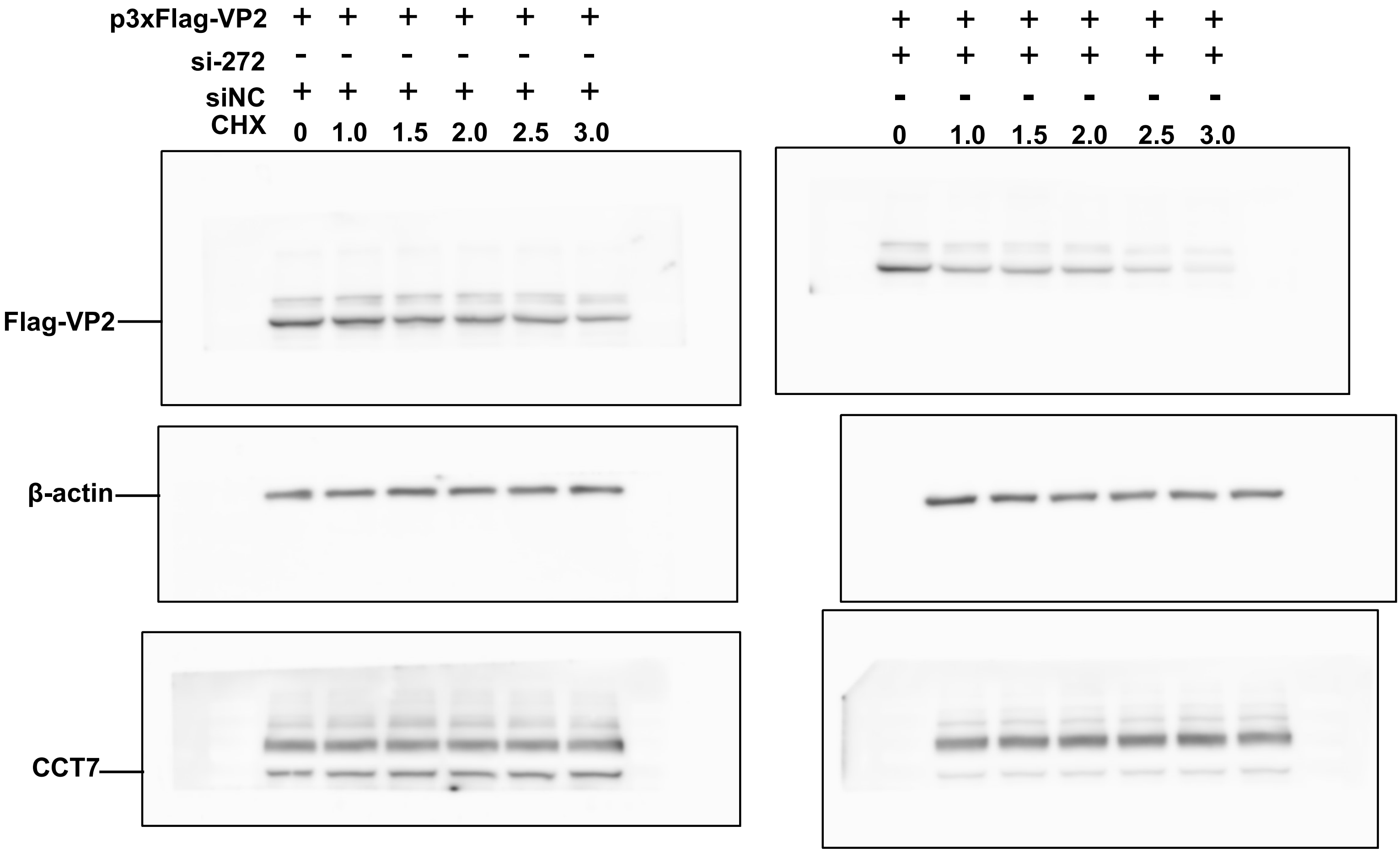

FIGURE 7A

The first biological repetition

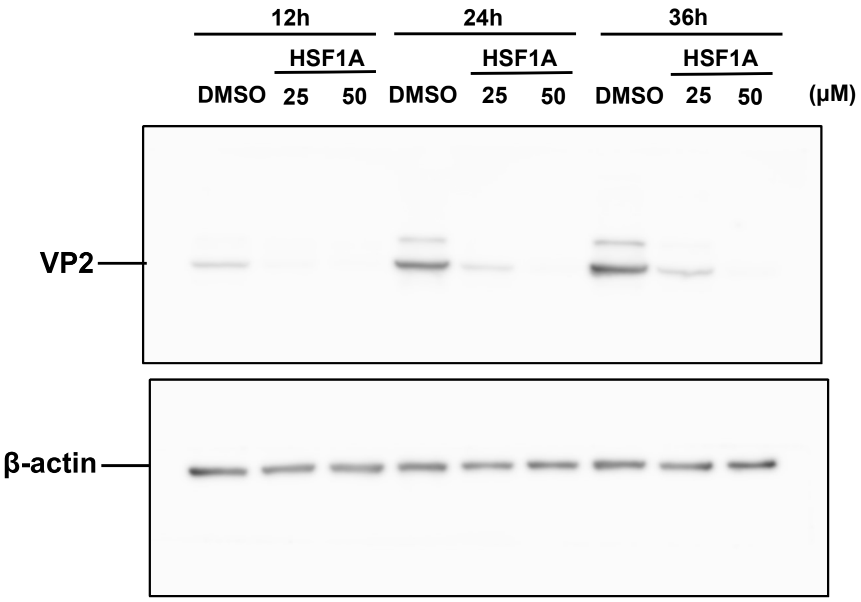

The second biological repetition

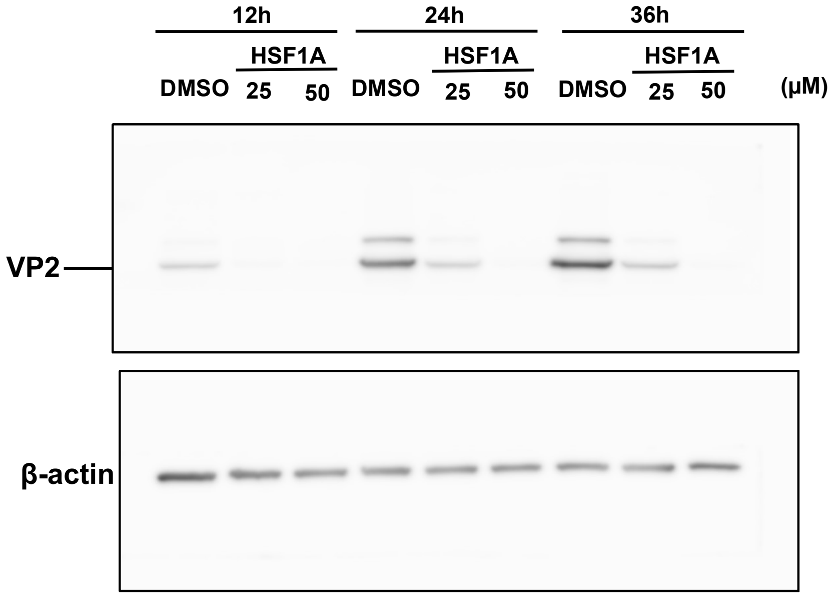

The third biological repetition,for drawing

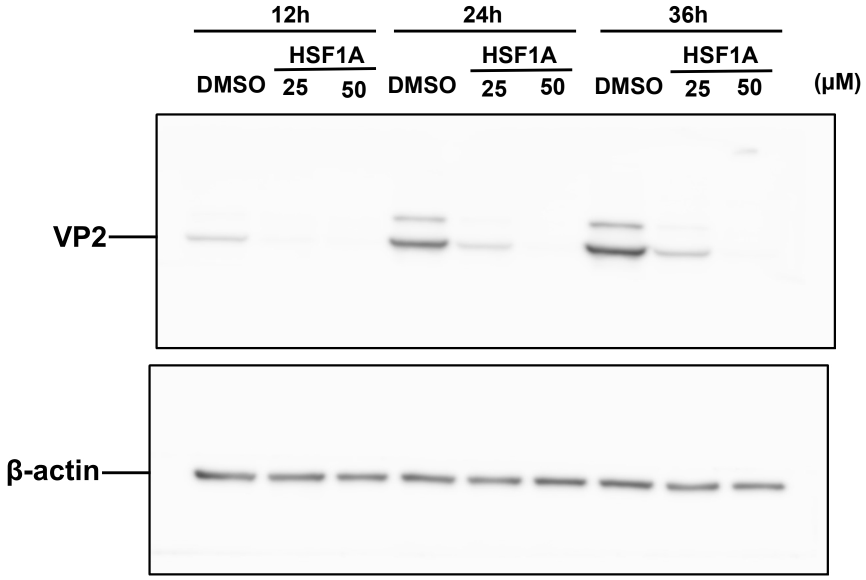

Supplement: Supplementary file 2 [file Data_Sheet_1.ZIP › Raw data/CCT7 WB original data.pdf]
